# Epithelial Sheet Folding Induces Lumen Formation by Madin-Darby Canine Kidney Cells in a Collagen Gel

**DOI:** 10.1371/journal.pone.0099655

**Published:** 2014-08-29

**Authors:** Sumire Ishida, Ryosuke Tanaka, Naoya Yamaguchi, Genki Ogata, Takeomi Mizutani, Kazushige Kawabata, Hisashi Haga

**Affiliations:** 1 Transdisciplinary Life Science Course, Faculty of Advanced Life Science, Hokkaido University, Sapporo, Japan; 2 Research Center for Cooperative Projects, Hokkaido University Graduate School of Medicine, Sapporo, Japan; University of Cambridge, United Kingdom

## Abstract

Lumen formation is important for morphogenesis; however, an unanswered question is whether it involves the collective migration of epithelial cells. Here, using a collagen gel overlay culture method, we show that Madin-Darby canine kidney cells migrated collectively and formed a luminal structure in a collagen gel. Immediately after the collagen gel overlay, an epithelial sheet folded from the periphery, migrated inwardly, and formed a luminal structure. The inhibition of integrin-β1 or Rac1 activity decreased the migration rate of the peripheral cells after the sheets folded. Moreover, lumen formation was perturbed by disruption of apical-basolateral polarity induced by transforming growth factor-β1. These results indicate that cell migration and cell polarity play an important role in folding. To further explore epithelial sheet folding, we developed a computer-simulated mechanical model based on the rigidity of the extracellular matrix. It indicated a soft substrate is required for the folding movement.

## Introduction

Lumen formation is required for embryonic morphogenesis [1]. Madin-Darby canine kidney (MDCK) cells form a luminal structure in three-dimensional (3D) culture environments. MDCK cells embedded in collagen gel form a cyst, retaining their apical and basolateral polarity. The addition of hepatocyte growth factor to the cyst leads to the formation of tubular extensions [2]. During lumen formation, the elimination of polarity causes the formation of abnormal luminal structures. The inhibition of integrin-β1 or Rac1 activity inverts cell polarity and perturbs lumen formation, and under these conditions, the inhibition of RhoA expression rescues normal polarity and luminal morphogenesis [3], [4]. Lumen formation is induced by overlaying an MDCK monolayer sheet with a collagen gel, a technique [5] that facilitates the reorganization of apical-basolateral polarity; however, lumen formation is prevented in the presence of antibodies that inhibit integrin-β1 activity [6], [7]. Further, constitutively-active and dominant-negative forms of Rac1 or RhoA influence lumen formation after a collagen gel overlay [8]. Taken together, these results indicate that integrins and small G proteins play essential roles in determining polarity and lumen formation.

Collective cell migration is an important activity of epithelial cells, especially in developmental morphogenesis *in vivo*. Examples include border-cell migration in *Drosophila*, lateral-line migration in zebrafish, and gastrulation in vertebrates [9], [10]. The collective migration of epithelial cells occurs in response to various extracellular stimuli such as signaling molecule gradients, substrate rigidity, and wound healing. In collective migration, a “leader cell”, which is a specialized cell with a large lamellipodium, often appears at the edge of an epithelial sheet and accompanies neighboring cells called “follower cells” [10]–[12]. However, it is unknown whether collective cell migration is involved in lumen formation.

Integrins and small G proteins regulate cell migration. Integrins form heterodimers of α and β subunits and interact with the extracellular matrix (ECM). Integrin-β1 interacts with collagen fibers and generates signals that control cell motility, proliferation, survival, and differentiation [13]. Small G proteins act as downstream components of the integrin signaling pathway and serve as key regulators of actin organization. Examples include the lamellipodial activity of Rac1 as well as filopodia formation and actomyosin contractility mediated by Cdc42 and RhoA, respectively [14]. Actomyosin is a complex of actin filaments and myosin II. Myosin II provides motor activity when Ser19, Thr18, or both of the myosin regulatory light chain (MRLC) are phosphorylated by the Rho-Rho kinase (ROCK) or CaM-MLCK pathways, or both [15], [16]. During cell migration, integrin-β1 localizes to the leading edge and interacts with the ECM [17]–[19] while Rac1 induces the extension of lamellipodia at the leading edge, and RhoA regulates the actomyosin contractile force through phosphorylation of the MRLC [16].

In the present study, we show that a collagen gel overlay induced epithelial sheet folding from the periphery that migrated inwardly, resulting in the formation of a luminal structure in a collagen gel. We demonstrate that these processes required integrin-β1, Rac1, and cell polarity. To explain the folding phenomena, we conducted a computational analysis that considered the rigidity of the surrounding substrate.

## Materials and Methods

### Cell culture and 3D collagen culture assay

MDCK cells were obtained from the Riken Cell Bank and maintained in Dulbecco's modified Eagles' medium (DMEM; Sigma-Aldrich, St. Louis, MO) containing 10% fetal bovine serum (FBS; Equitech-Bio. Inc., Kerrville, TX) and 1% antibiotic/antimycotic solution (Sigma-Aldrich). The cells were incubated in a humidified incubator at 37°C in an atmosphere containing 5% CO_2_. A 1.6 mg/ml collagen type I gel consisting of Cellmatrix type I-P (Nitta Gelatin Inc., Osaka, Japan) was used for the collagen overlay assay. First, an 8.0- or 12.5-mm radius glass dish was filled with 150 or 300 µl of the collagen gel, respectively, onto which trypsinized cells (1.0×10^3^ or 2.0×10^3^, respectively) were seeded. After culture for 4 or 5 days, a collagen gel solution (equal in volume to the first gel layer) was poured onto the cells and incubated for 30 min at 37°C and allowed to gel. The dish was then filled with culture medium. To observe gel deformation or the migration rate of each epithelial cell during lumen formation, an epithelial sheet was generated on a collagen gel. A plastic circular cylinder (1.5-mm radius) was glued to a 5-mm radius glass coverslip. A hole was introduced in the cover slip along the inside edge of the plastic cylinder. A 12.5-mm radius glass dish was filled with 300 µl of collagen gel. Before the dish was filled with culture medium, the plastic cylinder attached to the cover slip was placed onto the center of the collagen gel. After confirming that the cover slip adhered to the gel surface, culture medium was added around the cylinder. MDCK cells were then added to the cylinder and incubated overnight. The cylinder was removed gently to reveal an epithelial sheet on the collagen gel. The cells were overlaid with the collagen gel that was embedded with 2.0-µm latex beads (Polysciences, Inc., Warrington, PA) to allow observation of the deformation of the collagen gel induced by cellular contractile forces. Coverslips coated with 10% collagen type I (Cell matrix I-C, Nitta Gelatin Inc.) served as glass surfaces. For the collagen gel sandwich assay, the collagen solution was layered over the coverslip with a radius smaller than that of the glass dish. After the collagen gelled, the coverslip was gently placed onto the MDCK sheets, collagen-side down.

### Reagents

Monoclonal antibody (mAb) AIIB2 (the Developmental Studies Hybridoma Bank at the University of Iowa), Rac1 inhibitor II (Z62954982, Calbiochem, La Jolla, CA), and Y27632 (Sigma-Aldrich) were used to inhibit the activities of integrin-β1 [20], Rac1, and ROCK, respectively. Transforming growth factor (TGF)-β1 (#1210209 F2011, PeproTech, Inc., Rocky Hill, NJ) was used to alter apical-basolateral cell polarity. Roscovitine (Calbiochem) was used to inhibit cell proliferation. For fluorescence staining of F-actin, Alexa Fluor-488 phalloidin (Invitrogen, Carlsbad, CA) was diluted 1∶500. For immunofluorescence staining, antibodies used were as follows: anti-pig collagen (Monosan, The Netherlands); anti-phospho-MRLC (Thr18/Ser19) (diphosphorylated MRLC, PP-MRLC) rabbit IgG, Tri-Methyl-Histone H3 (Lys4) (C42D8) rabbit mAb for nuclei, and p-histone H3 (S10) rabbit mAb (p-Histone) (all from Cell Signaling Technology Japan, K.K., Japan); anti-gp135 (a gift from Dr. George Ojakian, Department of Cell Biology, SUNY-Downstate Medical Center) for gp135; rr1 (the Developmental Studies Hybridoma Bank at the University of Iowa) for E-cadherin; and AIIB2 for integrin-β1. The dilution of each antibody was as follows: collagen 1∶20, PP-MRLC 1∶100, nuclei 1∶4000, p-histone 1∶1000, gp135 1∶300, E-cadherin 1∶200, and integrin-β1 1∶100. Alexa Fluor-594-labeled goat anti-mouse IgG (H+L; Invitrogen), Alexa Fluor-594-labeled goat anti-rabbit IgG (H+L; Invitrogen), and Alexa Fluor-594-labeled donkey anti-rat IgG (H+L; Invitrogen) were used as secondary antibodies (10 µg/ml). DAPI was used to stain nuclei (a gift from Dr. Ushiki and Dr. Hoshi, Niigata University).

### Recombinant plasmid construction and transfection

A plasmid encoding the membrane trafficking domain of human H-Ras [21] and Azami-green (AG), which is a green fluorescent protein produced by *Galaxeidae* coral [22], was generated and designated phmAG1-H-Ras-CAAX. The H-Ras CAAX motif was then ligated to phmAG1-MCLinker (Takara Bio Inc., Shiga, Japan). The DNA sequence of the H-Ras CAAX motif was designed with the EcoRI and BamHI sites by using the following primer set: 5′-GATCCGGCTGCATGAGCTGCAAGTGTGTGCTCTCCTGAG-3′ (forward) and 5′-AATTCTCAGGAGAGCACACACTTGCAGCTCATGCAGCCG-3′ (reverse). The motif fragment was ligated into the phmAG1-MCLinker digested with EcoRI and BamHI. After constructing the AG-CAAX plasmid, MDCK cells on a plastic dish were transfected with the plasmid using Lipofectamine 2000 (Invitrogen). Colonies of transfected cells were selected using media containing G418 (Promega, Fitchburg, WI) and were harvested using a micropipette to establish the transgenic cell line MDCK-CAAX.

### Time-lapse imaging

Immediately after the gel overlay procedure, the dish was filled with culture medium and sealed with silicone grease to avoid exposure to air and changes to the pH of the medium. A phase-contrast microscope (TE300; Nikon, Tokyo, Japan) equipped with a 10× objective lens and an acrylic resin incubation box maintained at 37°C was used for time-lapse observations. Image-Pro software (Media Cybernetics Inc., Silver Spring, MD) was used to capture images every 5 min, and the images were edited to create movies. MDCK-CAAX cells were imaged using a confocal laser scanning microscope (TCS-SP5; Leica Microsystem CMS GmbH, Germany) coupled to a Leica DMI6000 CS microscope. Cells cultured with a collagen gel embedded with beads were imaged using a confocal laser scanning microscope (A1R Confocal Imaging System (Nikon)). The TCS-SP5 or A1R were equipped with a 63× or 60× objective lens, respectively, and maintained at 37°C. Images were captured at 20 min or 10 min intervals for TCS-SP5 or A1R, respectively.

### Analysis of migration velocity

**Figure 1:**
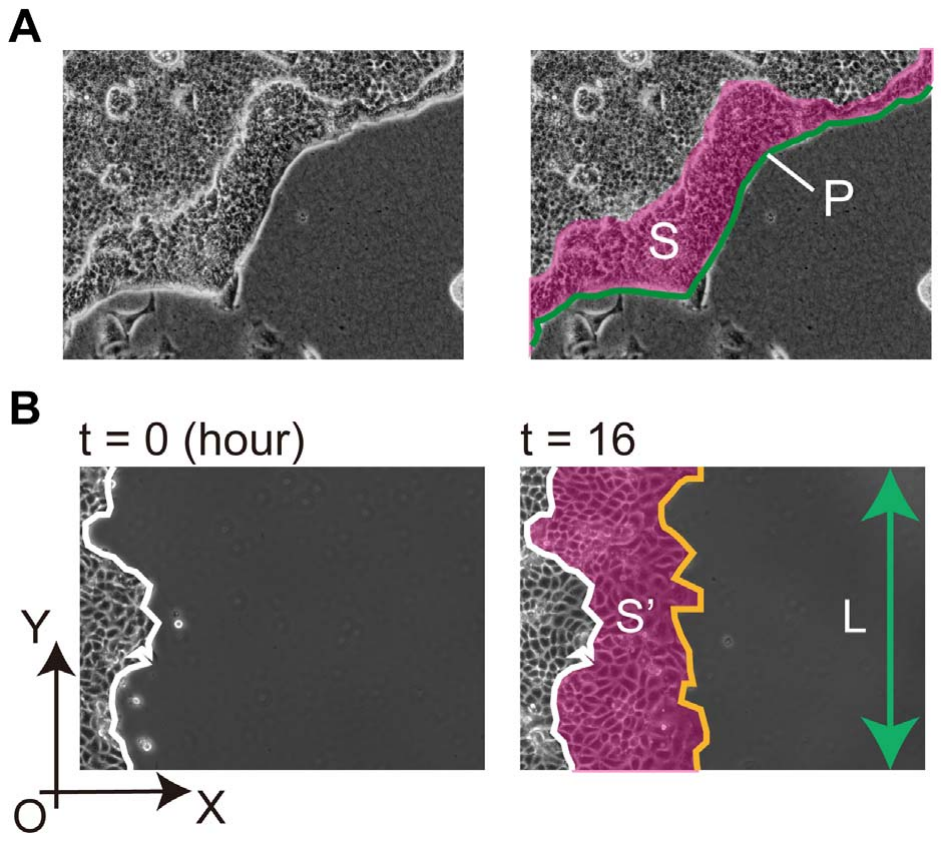
Measurement of the migrating area and the outer perimeter to determine migration velocity. *(A)* The image on the left represents the raw data and that on the right shows the measurement example. *S*, indicated by the pink area, represents the folded area. *P*, indicated by the green line, represents the outer perimeter of the epithelial sheet. *(B)* The white line represents the leading edge at the beginning of the observation. Two black arrows denote the *X*-axis and *Y*-axis, respectively, and *O* is the origin. The orange line denotes the leading edge at the indicated time point. *S′*, indicated by the pink area, represents the migrating area. *L*, indicated by the green arrow, represents length of the MDCK colony in the *Y*-axial direction.

To determine the migration velocity of epithelial cells, time-lapse images were acquired after overlay of the collagen gel. The sizes of the folded area (*S*) and the outer perimeter of the epithelial sheet (*P*) were calculated using Image Pro software (Fig. 1*A*). The average distance from the outer periphery to the leading edge was calculated using the equation *S*/*P*. The distance was plotted as a function of the observation time, a linear approximation was applied, and the slope was defined as the migration velocity. Inhibitors were added individually during lumen formation, after which the change of velocity was estimated. To calculate the migration velocity of epithelial cells on a glass surface, an epithelial sheet was formed on a collagen-coated glass coverslip using a cylinder. After the cylinder was gently removed, cell migration in the horizontal plane was observed. The area of migration (*S′*) and the length of the epithelial sheet in the vertical plane (*L*) were measured using Image Pro software (Fig. 1*B*). The average migration distance was calculated using the equation *S′/L*. The migration velocity was estimated by plotting the distance as a function of time.

### Immunofluorescence

Cells were fixed with 1% or 2% paraformaldehyde in phosphate-buffered saline (PBS) for 10 min for PP-MRLC or other proteins, respectively. The samples were permeabilized by incubation in 0.5% Triton-X100 in PBS for 10 min and blocked with 0.5% skim milk (Megmilk Snow Brand Co., Ltd., Hokkaido, Japan) in PBS for PP-MRLC or with 0.5% bovine serum albumin (Sigma-Aldrich) in PBS for other proteins. All samples were incubated with primary antibody overnight at 4°C for nuclei or at room temperature for other proteins. The appropriate secondary antibody and Alexa Fluor-488-conjugated phalloidin were then added for 3 h at room temperature. Cells were incubated with DAPI at 37°C for 1 h to stain nuclei. Images of fluorescent cells were captured using a confocal laser scanning microscope (C1 confocal Imaging System (Nikon) or A1R Confocal Imaging System).

### Collagen Zymography

The culture supernatant of MDCK cells was harvested during lumen formation and mixed with and equal volume of 2× loading buffer (0.5 M Tris-HCl, pH 6.8; 3% glycerol; 0.004% bromophenol blue). The samples were subjected to 0.25% collagen-7% SDS-polyacrylamide gel electrophoresis. After electrophoresis, gels were washed with renaturation buffer (2.5% Triton-X100; 54 mM Tris-base, pH 7.5; 200 mM CaCl_2_; and 200 mM NaCl) and incubated in a developing buffer (54 mM Tris-base, pH 7.5; 200 mM CaCl_2_; and 200 mM NaCl) at 37°C for 24 h. The gel was stained with Coomassie brilliant blue R250.

## Results

### A collagen gel overlay induced lumen formation by an epithelial sheet

**Figure 2:**
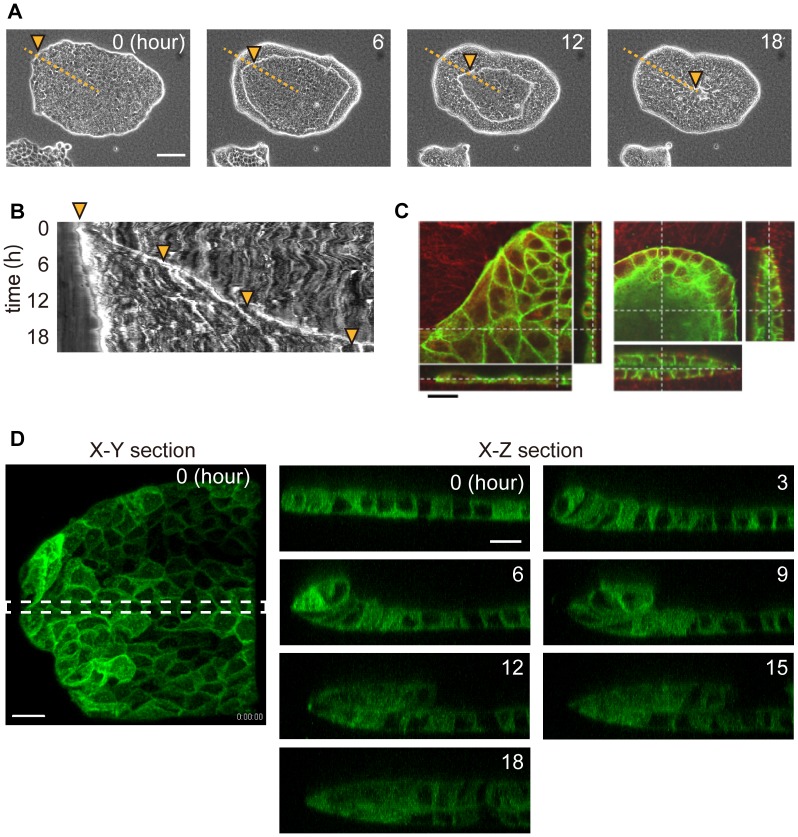
Epithelial sheets formed luminal structures by folding after the collagen gel overlay. *(A)* Time-lapse images of an epithelial colony after the collagen gel overlay. Images were acquired using a phase contrast microscope. Numbers in the images represent the relative time from the start of the observation. The orange dotted line corresponds to the horizontal axis of the kymograph in Fig. 2*B*. The orange arrowheads indicate the position of the leading edge of the MDCK sheets, and they correspond with the kymograph. Bar = 100 µm. *(B)* Kymograph of epithelial folding. *(C)* Fluorescent images of F-actin (green) and collagen (red). Bar = 20 µm. *(D)* Images were acquired using a confocal fluorescence microscope. Green represents AG-CAAX (plasma membrane domain). *X-Z* sectional views were merged from the area indicated by the dotted line in an *X-Y* sectional view. Bar = 20 µm.

An MDCK epithelial sheet was cultured on a collagen gel and overlaid with another collagen gel. Immediately after the overlay, the MDCK cells began to fold from the periphery and migrated collectively toward the center of the epithelial sheet at a constant velocity, forming a lumen structure (Fig. 2*A–C*, Movie S1). Folding occurred only when the upper and lower substrates were collagen gels, and they were not induced if the upper or lower substrate was a collagen-coated glass coverslip (Fig. S1).

To obtain high-resolution images of cell morphology during lumen formation, we performed 3D imaging of viable MDCK-CAAX cells. The cells migrated from the periphery toward the center of the epithelial sheet and maintained the sheet structure, which finally formed the luminal structure (Fig. 2*D*, Movie S2).

The luminal structure maintained the initial shape of the epithelial sheet before and after the gel overlay. For example, the epithelial sheets were cut in an arbitrary shape using a micromanipulator (Fig. S2, Movies S3, S4, S5, S6). When the epithelial sheet was shaped into letters such as “L,” “O,” “V,” or “E,” the luminal structure retained its shape after the gel overlay.

### Cells near the leading edge contributed to folding

We determined whether all of the cells within the colony migrated during the folding of MDCK cell sheets by measuring the cells' velocity field. Mosaic colonies were formed by mixing fluorescent cells with nonfluorescent cells that were cultured on a collagen gel. After the collagen-gel overlay, folding was monitored simultaneously using phase contrast and fluorescence microscopy (Fig. S3). The cells near the leading edge migrated at the same rate as the leading edge moved ahead. In contrast, the cells distant from the leading edge rarely migrated.

### Inhibition of either integrin-β1 or Rac1 delayed lumen formation

**Figure 3:**
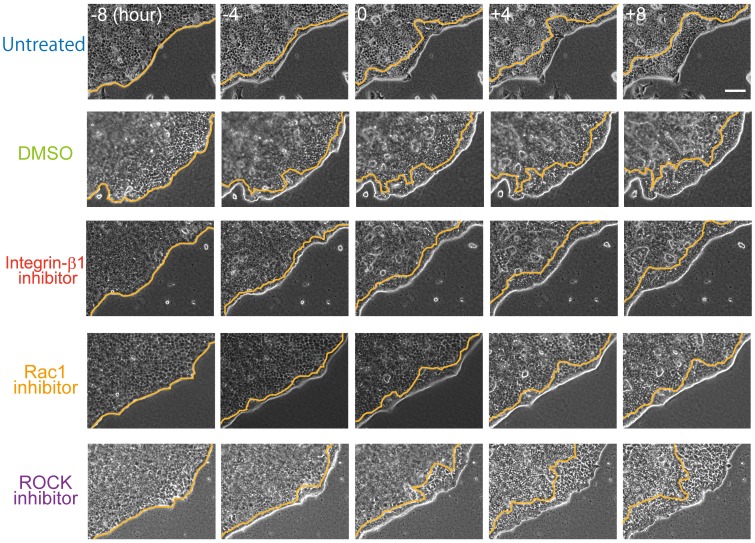
Temporal sequence of folding in the presence of an inhibitor. Integrin-β1 inhibitor (AIIB2, 100 ng/ml); Rac1 inhibitor (Z62954982, 100 µM), ROCK inhibitor (Y27632, 10 µM); DMSO was used as a control for the Rac1 inhibitor. Each reagent was added at time zero. Orange lines represent the leading edges of the folding sheet. Numbers in the images denote the observation time (h). Bar = 100 µm.

To determine the contribution of cell migration to lumen formation, we treated cells with AIIB2, Z62954982, or Y27632 to inhibit integrin-β1, Rac1, or ROCK, respectively. To minimize experimental error, large epithelial sheets were prepared so that folding initiated from relatively straight periphery (Fig. 3). Compared with the control cells, the velocity of cell migration decreased in the presence of AIIB2 or Z62954982. In contrast, cells migrated faster compared with controls in the presence of Y27632.

**Figure 4:**
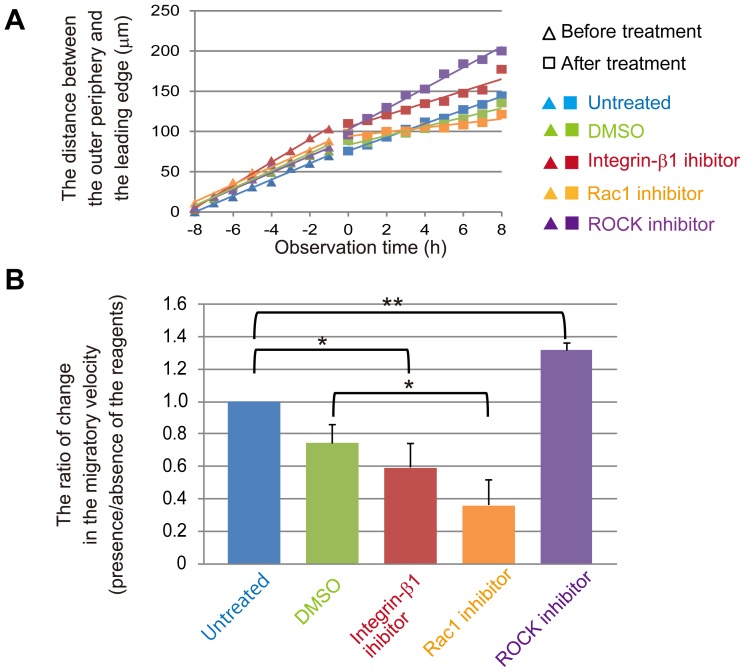
Inhibition of either integrin-β1 or Rac1, but not ROCK, delayed folding. *(A)* The scatter plot shows the migration distance from the outer periphery to the leading edge for each treatment. Observation time corresponds to that in Fig. 3. The equation used to calculate the average distance is described in Materials and Methods. The mean values of three independent experiments are shown. *(B)* Histogram indicating the mean ratio of the migration velocity in the presence of inhibitors. The ratio was calculated by dividing the migration velocity after inhibitor treatment by the velocity before treatment. The mean values are shown with SD (shown as error bars) from three independent experiments, *p<0.05, **p<0.01.

The distance between the outer periphery and the leading edge of the fold was plotted as a function of time (Fig. 4*A*). Cell migration was observed for 8 h before and after inhibitor treatment. The migration velocity was calculated from the slope of the linear approximation of the curve, and the ratio of change in migration velocity was estimated (Fig. 4*B*).

To confirm whether Y27632 inhibited ROCK activity, we performed immunofluorescence staining of PP-MRLC in MDCK cells on a collagen gel (Fig. S4). In untreated cells, PP-MRLC was localized to the smooth periphery of the colonies but was not detected when the cells were treated with Y27632 (Fig. S4 *A*). PP-MRLC was also detected at the periphery of leader cells. In contrast to the smooth edge, PP-MRLC was detected in leader cells in the absence or presence of Y27632 (Fig. S4 *B*). Further, migration of leader cells was not prevented by Y27632 (Fig. S4 *C*).

In the experiments described above (Fig. 4), the inhibitors were added 8 h after folding commenced. To determine whether integrin-β1, Rac1, and ROCK activities contribute to the initiation of folding, inhibitors were added for at least 30 min before the gel overlay. The ratio of the velocities of treated to untreated cells was estimated using the same methods described above (Fig. S5 *A, B*). Consistent with the data shown in Fig. 4, AIIB2 and Z62954982 decreased the velocity of migration, and Y27632 had no significant effect.

### The flattening of epithelial cells increased the surface area of the sheet during lumen formation

The epithelial colonies maintained the initial area of the sheet during folding (Fig. 2*A*), causing the surface area of the epithelial monolayer to double in size after lumen formation. To determine how the cells increased the surface area, we first observed whether the cells proliferated during folding. Histone-H3 Ser10 is phosphorylated during mitosis [23]. Few fluorescent cells were stained by the anti-p-histone antibody midway during folding (Fig. S6 *A*). The cells were next treated with the inhibitor of cell division roscovitine, which did not prevent folding (Fig. S6 *B*). A *Z*-sectional view of the folding epithelial colony revealed that the cells in the top layer became flatter compared with those in the bottom layer (Fig. S6 *C, D*), suggesting that during lumen formation, the surface area of epithelial colonies was increased by the flattening of cells in the top layer.

### Disrupting cell polarity inhibited folding of the epithelial cell sheet

**Figure 5:**
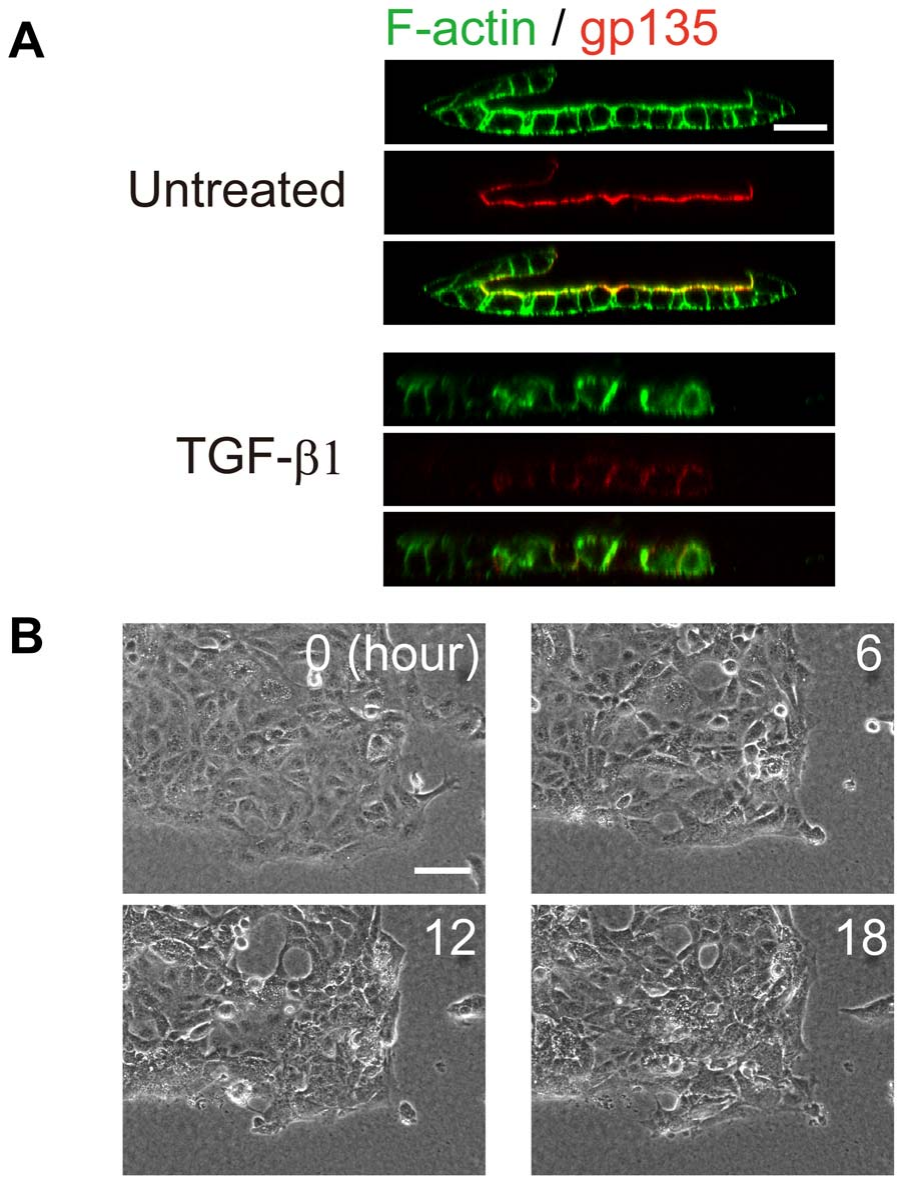
Disruption of cell polarity induced by TGF-β1 treatment prevented folding. *(A)* F-actin (green) and gp135 (red) fluorescence. Cells were treated with TGF-β1 (1.5 ng/ml) for 2 days before fixation. Bar = 25 µm. *(B)* Time-lapse images of polarity-disrupted epithelial colonies after the gel overlay. Numbers in the images represent the observation time (h). Bar = 100 µm.

We next focused on the contribution of cell polarity to epithelial sheet folding. Immunohistochemical analysis of the apical marker gp135 indicated that apical-basolateral polarity was maintained during epithelial sheet folding (Fig. 5*A*) and that the apical surface of the cells always faced the inside of the epithelial sheet. When MDCK cells were treated with TGF-β1, which disrupts cell polarity [24], the sheet did not fold in response to a gel overlay (Fig. 5*A*), but instead, moved randomly and did not form luminal structures (Fig. 5*B*, Movie S7). TGF-β1 treatment significantly inhibited epithelial sheet folding (Fig. S7 *A, B*), which was indicated by the distribution of gp135, integrin-β1, and E-cadherin over the entire cellular surface, indicating that cell polarity was disrupted (Figs. 5*A* and S7 *C, D*).

### Integrin-β1 localized to the apical surface at the colony edge before the gel overlay

To observe how cells at the periphery of the colony initiate folding, we focused on integrin-β1, because pretreating cells with AIIB2 delayed lumen formation (Fig. S5). We found that disrupting the localization of integrin-β1 prevented folding (Fig. S7 *C*). Therefore, we reasoned that the polarized localization of integrin-β1 before the gel overlay played a key role in folding. We found that integrin-β1 localized to the entire cell surface only at the periphery of the colony (Fig. S8).

### During lumen formation, epithelial cells deformed a collagen gel through MRLC activity

Mechanical force plays important roles in morphogenesis [25], [26]; therefore, we asked whether MDCK cells generate a mechanical force during folding. To answer this question, latex beads were mixed with a collagen gel, and lumen formation was observed. The beads moved by approximately 10.5 µm along the direction of cell migration on the *X-Y* plane, but rarely along the *Z*-axis, for 12 h after the gel overlay (Fig. S9 *A*).

Actomyosin generates a contractile force through the phosphorylation of MRLC [15]. Therefore, we determined the localization of actomyosin within the colony. The images of fluorescently labeled F-actin and PP-MRLC showed that actomyosin localized to the leading edges of the folding MDCK sheet (Fig. S9 *B*), and the localization of PP-MRLC revealed that leader cells were present in some regions of the leading periphery during folding. Because leader cells exert a traction force on substrates through PP-MRLC [27], these results suggest that MDCK cells generated a traction force at the leading edge of cell migration during lumen formation.

### MDCK cells degraded the collagen gel during lumen formation

In addition to traction force, the degradation of the ECM plays an important role in cell migration through a 3D matrix. Because MDCK cells secrete matrix metalloproteinase (MMP) isoforms that degrade collagen during tubulogenesis [28], [29], we determined whether degradation of the ECM contributed to folding. Immunofluorescence staining of collagen detected a collagen gel-free region between collagen layer overlay and the bottom cell layer during folding (Fig. S10 *A*). We performed collagen zymography to determine whether MDCK cells degraded collagen. Considering the molecular masses of MMPs [30], the result reveals that MDCK cells likely secreted MMP-8 during the folding phenomena (Fig. S10 *B*). To determine whether the degradation of the ECM contributed to folding, MDCK cells were treated with the MMP inhibitor GM6001 and overlaid with the gel. GM6001 treatment reduced the size of the collagen gel-free space, flattened the folding epithelial sheet, and decreased migration velocity (Fig. S10 *C*, *D*). These results suggest that the degradation of the ECM helps cells migrate smoothly through the collagen matrix. In addition to the traction force generated by MRLC, ECM degradation also plays an important role in gel overlay-induced lumen formation.

### When sandwiched between collagen gels, epithelial cells did not migrate to the space around the colony

The inhibition of MMP activity by GM6001 reduced the size of the gel-free space, although the lumen still formed. This indicates that folding was not a variation of cell migration on a planar surface. In the collagen gel system, the cells degrade the surrounding substrate by secreting MMP, create the space between the top and bottom cell layer, and migrate on the surface of the gel layer. To determine whether folding was caused by the migration of cells to the collagen gel-free space, we observed MDCK cells by using a collagen gel sandwich assay. In the collagen gel sandwich assay, gelled collagen is layered on the colonies, in contrast to the collagen gel overlay in which the collagen solution gels after it is poured over the cells. By changing the order of gelation, the sandwich methods provide the cells with a gel-free region between the upper and lower gels (Fig. S11). Despite the presence of a gel-free space surrounding the colony, the MDCK cells did not spread to the free space but migrated toward the center of the colony. Therefore, the collagen gel sandwich assay indicates that folding was not a variant of 2D cell migration.

### A soft substrate is required for folding

**Figure 6:**
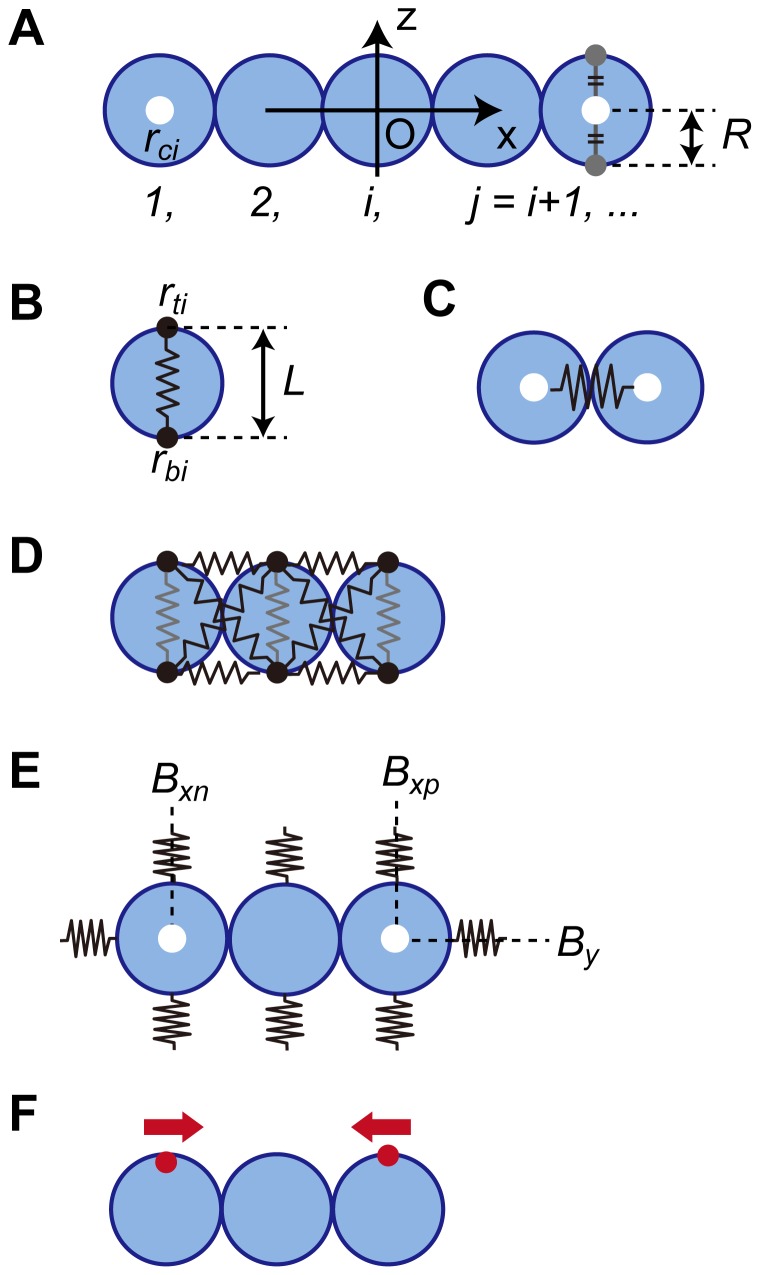
Definitions and parameters of the simulation. *(A)* 2D model of an MDCK cell: An MDCK cell is illustrated as a blue circle with a radius of *R*. *X-Z* section of an MDCK sheet is represented as a chain of the circles. The origin is the center of the chain. The circles are numbered in order from the negative edge of the chain, represented as *i* in subscripts; *j = i*+1. A white dot indicates the center of *i* ^th^ blue circle, represented by *r_ci_*. *(B)* Force causing random motion: Two black dots on the periphery of a circle move randomly on the *X-Z* plane. Their positions are represented as *r_ti_* or *r_bi_*; *t* or *b* in subscript indicate top or bottom, respectively. The two dots are connected by a spring with a natural length of *L*, (black line). *(C)* The repulsive force is represented by a spring between adjacent circles. *(D)* Shear tolerance: composed of pairs of parallel and crossed springs (black) between neighboring circles. *(E)* Elastic force generated by the surrounding substrate: represented by springs on the surface of the circles. *B_x_* or *B_z_* is initial *X* or *Z* coordinate of *r_ci_*, respectively. The subscripted *p* or *n* indicates *B_x_* of the positive or negative edge, respectively. *(F)* Migration force: represented as red dots that apply force to the coordinate origin. This force is applied to the *r_ti_* at the edges.

The rigidity of the ECM changes cell behaviors such as migration and adhesion [11], [31], [32]. To determine how a glass substrate prevented folding (Fig. S1), we focused on the rigidity of the substrate. To investigate the mechanical contribution of the substrate to folding, we performed a computational simulation by building a 2D mathematical model of a vertical cross section of an epithelial sheet. The parameters used in the model are shown in Fig. 6. Fig. 6*A* shows the 2D model of the MDCK cells and several definitions of the simulation (described in detail in Protocol S1). Each blue circle represents an MDCK cell. A chain of blue circles represents the vertical cross section of an MDCK sheet. The center of the chain is defined as the origin of the *X-Z* coordinate. The white dot represents the position of the center of the blue circle. Fig. 6, *B–F* shows the five parameters used in the simulation. First, a force causing random motion is applied to the two points on the circumference of a circle (black dots in Fig. 6*B*). The motion of the points is restricted on the *X-Z* plane. The force causing random motion is defined by Eq. 1 (all equations are explained in Protocol S1). Those positions are connected by a spring (illustrated as a black line in Fig. 6*B*, Eqs. 2*A* and 2*B*). Note that the first positional relationship between top and bottom dots is constant, just as apical-basolateral polarity is maintained during folding (Fig. 5*A*). Second, Fig. 6*C* represents the repulsive force applied between the blue circles (Eq. 3). Third, shear tolerance is applied to maintain the monolayer. The blue circles are restricted by the springs, which are represented in black in Fig. 6*D*. The dynamics of the parallel springs are described by Eqs. 4*A* and 4*B*, and one of the crossed springs is described by Eqs. 5*A* and 5*B*. Fourth, Fig. 6*E* represents the rigidity of the substrate. During folding, cells adhered to surrounding ECM without a space (Fig. 2*C*) and received the repulsive force from the substrate. The springs in Fig. 6*E* around the blue circles represent the repulsive force (Eq. 6). Last, the migratory force is applied as a driving force of cell migration after the gel overlay (Fig. 6*F*, Eq. 7). Further, assuming that the viscosity of the cellular environment is sufficiently high, inertia can be ignored, and the velocity is proportional to the force [33]. Together, the dynamics of the circle position are described by Eqs. 8*A* and 8*B*.

**Figure 7:**
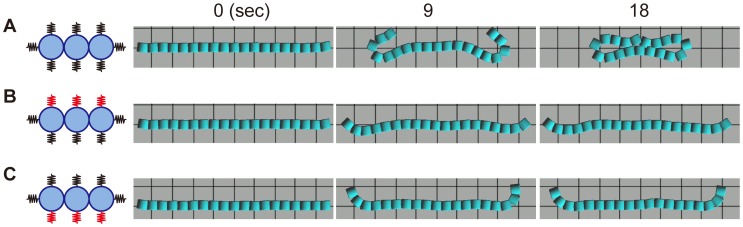
Temporal images of the computational simulation of epithelial sheet folding. The numbers above the images show relative time (s) from the start of modeling. *(A)* As a control, the parameters are modulated appropriately. *(B)* The rigidity of the upper substrate (red springs, the parameter in Fig. 6*D*) is increased. *(C)* The rigidity of the lower substrate (red springs) is increased.

By modulating these parameters appropriately, the model simulated folding (Fig. 7*A*, parameter values are presented at the end of Protocol S1). The monolayer buckled during folding, which did not occur in the MDCK cell sheet. This may be explained by the model's lack of a parameter for cell-substrate adhesion. Adding this parameter to the model is a challenge for the future.

We next increased the rigidity of the substrate to observe the contribution of substrate rigidity. The rigidity of the collagen gel here is approximately 0.6 kPa according to the results of another study [34]. The rigidity of a glass substrate (2–4 GPa) is virtually infinitely greater than that of the gel [32]. Therefore, we increased the spring constant to the maximum value for a rigid substrate and observed the shape of the monolayer. When the rigidity of the top or bottom spring was increased, the monolayer did not fold (Fig. 7*B, C*). These results indicate the rigidity of the surrounding substrate plays important roles in the folding of MDCK cells after the gel overlay.

## Discussion

Here we demonstrate that a collagen gel overlay induced the folding of an epithelial sheet from its periphery to form a luminal structure in a collagen gel. Although the migration of individual cells did not differ in a gel overlay or on a glass surface (Fig. S12) [35]–[37], folding did not occur when the upper or lower substrates were glass coverslips (Fig. S1). The rigidity of a substrate changes various cellular behaviors [11], [31], [32], [38]. For example, in MDCK cell cultures, directional collective migration is induced when the cells are cultured on a soft collagen gel [11]. We show here that similar collective migration occurs during lumen formation after the collagen gel overlay. Peripheral cells of the epithelial sheet migrated collectively toward the center of the colony after folding. Although these results indicate that a collagen gel provides a soft substrate for the collective migration of cells to form a luminal structure, the molecular mechanism is unknown.

Inhibitor treatment revealed that integrin-β1 or Rac1 activity, but not that of ROCK, contributed to cell migration velocity during lumen formation. These results are consistent with those of a previous study showing that ROCK inhibition enhances the migration of epithelial cells and fibroblast [39]. In addition, these results indicate that cell migration is essential for epithelial sheet folding and lumen formation, because integrin-β1 and Rac1 are crucial factors for cell migration, whereas ROCK contributes to the directional persistence of migration [40].

Cell polarity is important for epithelial sheet folding that forms a lumen. Our immunofluorescence study demonstrates that apical-basolateral polarity was maintained during lumen formation after a gel overlay. This result contradicts a previous study regarding lumen formation by MDCK cells cultured on a glass substrate in which the reorganization of cell polarity was observed after a collagen gel overlay, and cell migration was not observed [6]. These data suggest that the maintenance of cell polarity and cell migration are indispensable for lumen formation induced by folding of an epithelial sheet.

The observation of the velocity field during folding reveals that the cells around the migrating edge moved faster than those located elsewhere (Fig. S3). A similar velocity field is observed in cell migration during wound healing where the cells around the wound edge migrate faster than those farther from the edge [41]. The structure of the leading edge of migration is also similar. Folding and wound healing are characterized by actomyosin fibers and leader cells at the leading edge [42]. In contrast, apical-basolateral cell polarity shows a variable phenotype, which is lost in wound healing [43] but maintained during folding (Figs. 5 and S8 *C, D*). These data suggest that maintenance of cell polarity is a specific component of folding and its further study may provide insights into the molecular mechanisms of lumen formation after the application of a gel overlay. We do know from our present study that the disruption of cell polarity by TGF-β1 prevented lumen formation, but how cells maintain polarity is unknown. The regulation and maintenance of apical-basal polarity in epithelial cells by the Par3/Par6/aPKC complex [44] suggests an avenue of investigation for future studies. For example, silencing the complex may reveal in detail the role of maintained cell polarity in folding.

When gel sandwich assay provided free space around the peripheral cells, the cells did not spread out but folded inwardly (Fig. S11). This indicates that folding did not occur due to migration toward the matrix-free area. Because the peripheral cells faced the gel-free space between the top and bottom gel layers in the sandwich assay, whether apical-basolateral polarity was established at the edge should be considered. According to the results of previous studies, proper polarity of multicellular clusters is maintained while cell-ECM adhesion is prevented [2], [4]. Therefore, apical-basolateral polarity was possibly maintained even if the outer periphery of the MDCK colony did not adhere to the substrate. To investigate the cell polarity during gel sandwich assay, live-cell imaging of cells expressing GFP-integrin-β1 will be used.

Integrin-β1 localized to the apical surface at the periphery of the MDCK sheet before the gel overlay, and pretreatment with AIIB2 decreased the velocity of folding (Figs. S5 and S8). These data indicate that integrin-β1 is important for the initiation of lumen formation. Integrin-β1 is activated by adhesion to the ECM and promotes cell migration [13], suggesting that apical integrin-β1 helps detect the collagen gel overlay and immediately initiates migration. Therefore, the presence of integrin-β1 on the apical surface may play a key role in initiating folding along the periphery of the sheet.

Integrin-β1 plays an essential role in the initiation of collective migration *in vivo* [45], [46]. For example, cell migration during gastrulation in frogs is similar to folding studied here. Thus, the sheets of mesodermal and endodermal precursors roll into the blastopore, reverse the direction of migration toward the animal pole, and migrate on the surface of the prefolded cell layer via radial intercalation [45]. The adherence of integrin-β1 to the ECM is required in the early stage of gastrulation [46]. Future studies on the downstream effectors of integrin-β1, such as paxillin or FAK [13], may provide further insights into the role of integrin-β1 in the initiation of folding.

Three-dimensional imaging of cells in a matrix embedded with beads revealed that the matrix was mostly deformed along the direction of migration of the cells and rarely in the *Z*-axial direction. Immunofluorescent staining of PP-MRLC showed that cells generated a traction force at the leading edge of folding movement (Fig. S9). These results indicate that the cells generated a migratory force, which was used as the parameter in our simulation (Fig. 6*F*), and that a *Z*-axial force may not be produced.

The computational simulation of folding yielded results similar to those acquired from our experiments using MDCK cells, except for the buckling of the bottom layer. This discrepancy may be explained by the absence of a parameter for cell-substrate adhesion in the simulation, which was omitted to simplify the model. Future studies will include this parameter to improve the mechanical model of folding.

Consistent with the folding of the MDCK sheet, the computer simulation showed that a rigid substrate prevented folding. Because we did not use exact values for all of the parameters, further analyses are required, such as measuring the deformation volume of the ECM, the traction force during cell migration, and cell-cell adhesion force.

Although there are no reports of folding of an epithelial sheet *in vivo*, the manipulation of lumen formation *in vitro* is a promising method for tissue engineering. Here we succeeded to generate arbitrarily shaped tubes in a collagen gel (Fig. S2). If this method can be applied to vascular endothelial cells, then any branched blood vessel structure with an arbitrary shape can be formed and utilized for transplants.

In summary, a collagen gel overlay induced the integrin-β1 and Rac1-mediated folding of MDCK epithelial cells cultured on a collagen gel to form a luminal structure. Apical-basolateral polarity was maintained during the entire process. A computational approach, which considered stiffness of the surrounding substrate, simulated the folding of an epithelial sheet. Although the molecular mechanism of folding is unknown, the collagen gel overlay method shows great potential for tissue engineering.

## Supporting Information

Figure S1 **Folding movement did not occur on a glass substrate.** *(A)* Time-lapse images of epithelial cell colonies cultured on a collagen-coated glass and overlaid with a collagen gel. Bar = 100 µm. *(B)* Time-lapse images of epithelial cell colonies cultured on a collagen gel and overlaid with a collagen-coated glass cover slip. Bar = 100 µm. Each observation was started immediately after the overlay. Numbers represent the relative time (h) from the start of the observation.
(TIF)

Figure S2 **Lumen formation occurred regardless of the initial shape of the epithelial sheets.** *(A)* Time-lapse images of epithelial sheets cut in arbitrary shapes. Each observation was started immediately after the collagen gel overlay. Orange lines represent the leading edge of the cell migration. Numbers denote the observation time (h). *(B)* Detection of F-actin fluorescence in the “L”-shaped structure shown in Fig. S2 *A*. Images (1–3) are enlargements of indicated areas in the leftmost image.
(TIF)

Figure S3 **The cells near the leading edge migrated faster than those more distant.** Simultaneous phase contrast (top row) and fluorescence microscopy (bottom row) of migration. Times (h) are indicated in each panel. The colony is a mosaic of fluorescent (MDCK-CAAX cells) and nonfluorescent cells. The lines represent the leading edge of the folding movement. The arrows and arrowheads chase the cells at the leading edge or within the colony, respectively. Numbers indicate the relative times (h) from the start of the observation. Bar = 100 µm.
(TIF)

Figure S4 **Treatment with a ROCK inhibitor induced dephosphorylation of MRLC at the edge of colonies, but in the leader cells.** F-actin (green) and PP-MRLC (red) fluorescence of MDCK sheets cultured on a collagen gel. *(A)* Smooth edge. ROCK inhibitor (Y-27632, 10 µM) was applied for 30 min before fixation. Bar = 25 µm. *(B)* Leader cells. The cells were treated overnight with Y-27632 before fixation. The white arrowhead points to a leader cell. Bar = 25 µm. *(C)* Temporal imaging of a leader cell migrating on a collagen gel. ROCK inhibitor (Y27632, 10 µM) was added at time zero. The orange arrowheads point to the leader cells. Numbers indicate the observation time (h). Bar = 100 µm.
(TIF)

Figure S5 **Inhibition of either integrin-β1 or Rac1 but not ROCK, delayed early folding.** *(A)* The scatter plot shows the migration distance from the outer periphery to the leading edge for each treatment. Inhibitors were added at least 30 min before gel the overlay. The collagen solution was mixed with the indicated inhibitor and layered over the MDCK cells. Immediately after the gel formed, the observation started and continued for 16 h. The equation used to calculate the average distance is described in Materials and Methods. The mean values of at least three independent experiments are shown for untreated or cells treated with Y27632. The data acquired using the other reagents represent one experiment. *(B)* Histogram indicating the mean ratio of the migration velocity with or without inhibitors. The ratio is calculated by dividing the migration velocity of inhibitor-treated colonies by the velocity of untreated colonies. Shown are the mean values and SD (shown as error bars) from three independent experiments using Y27632. There was no significant difference in migration velocity between untreated and treated cells.
(TIF)

Figure S6 **The basal area of epithelial colonies increased by cell flattening.** *(A)* Epithelial sheets stained with DAPI (blue), and antibodies against p-histone (red) and F-actin (green) during folding. Red lines represent the planes from which the sectional views were generated. Bar = 50 µm. *(B)* Time-lapse imaging of roscovitine-treated (100 µM) epithelial colony after the gel overlay. Roscovitine was added immediately after the gel overlay. Numbers indicate observation times (h). The Orange line indicates the leading edge of folding. Bar = 100 µm. *(C)* The *Z* section of the image of F-actin fluorescence during folding. The blue and red arrowheads indicate flattened and columnar cells, respectively. Bar = 25 µm. (*D*) The ratio of flat to columnar cells in the colony before and after the gel overlay. After the gel overlay, the cell in the lower layer and those in the upper layer were counted separately. Cells were categorized as “flat” when the width was greater than height in the *Z* section. The mean values and SD (error bars) of 20 cells from two independent experiments; *p<0.05.
(TIF)

Figure S7 **TGF-β1 treatment prevented lumen formation.** *(A)* Categorization of folding and unfolding epithelial sheets. F-actin and nuclei were stained green and red, respectively. Cells were categorized as “folding type” when a space was observed between the upper and the lower layers of the epithelial sheet in the *Z* section of fluorescent images. Bar = 25 µm. *(B)* The ratio of folding to non-folding cells in the presence or absence of TGF-β1. The mean values are shown with SD (shown as error bars) from four independent experiments; *p<0.02. *(C–D)* Immunofluorescence of integrin-β1 or E-cadherin in untreated or TGF-β1-treated MDCK cells fixed 8 h after the gel overlay. The merged images with F-actin are also shown. Bar = 25 µm.
(TIF)

Figure S8 **Integrin-β1 localized to the apical surface at the periphery of the MDCK colony.** Integrin-β1 immunofluorescence (red) of MDCK cells on a collagen gel. The merged images with F-actin are also shown. The orange arrowheads point to the apical integrin-β1. Bar = 25 µm.
(TIF)

Figure S9 **MDCK cells deformed the collagen gel during lumen formation.** *(A)* 3D time-lapse images of MDCK cells within a latex bead-containing collagen gel. Images were acquired using the reflection interference mode of a confocal fluorescence microscope. The observation was started 30 min after the collagen gel overlay. Numbers denote the relative time from the start of the observation. The orange arrowhead points to the position of the beads at 0 h. Four beads were tracked in one experiment. Bar = 25 µm. *(B)* F-actin (green) and PP-MRLC (red) immunofluorescence in MDCK cells during lumen formation. Sectional views along the red lines are shown. The orange arrowhead points to a leader cell. Bar = 50 µm.
(TIF)

Figure S10 **MDCK cells degraded the collagen gel.** *(A)* Collagen (red) and F-actin (green) immunofluorescence in the MDCK colony during lumen formation. MDCK cells were fixed 6 h after the gel overlay. Red lines indicate the plane from which the sectional view was generated. Orange arrowheads point to the region between the upper collagen layer and the lower cell sheet that did not contain collagen. Bar = 20 µm. *(B)* Collagen zymography of the culture supernatant from MDCK cells under the gel. The black arrows point to the MMP bands that migrated at positions consistent with those of the precursor (upper) active (lower) forms of MMP-8. *(C)* Collagen (red) and F-actin (green) immunofluorescence in an MDCK colony treated with DMSO or GM6001 (30 nM) *Z* sections. The cells were fixed 15 h after the gel overlay. Orange arrowheads point to regions without the collagen gel. Bar = 25 µm. *(D)* Time-lapse observation of MDCK cells in the presence or absence of GM6001, which was added after the collagen gel overlay. The orange line represents the leading edge of the migrating sheet. Numbers indicate the relative time from the start of the observation. Bar = 100 µm.
(TIF)

Figure S11 **MDCK cells under the collagen gel did not migrate to collagen-gel free space.** Interference reflection images of an MDCK sheet 11 hours after the gel overlay. The cells were fixed 15 h after the gel overlay. Sectional views were generated from the red lines. The orange arrow in the right column of the image on the left points to the cells that migrated to the upper layer. The image in the right panel is an overexposure. Orange arrowheads point to the collagen gel-free space between collagen layers. Bar = 50 µm.
(TIF)

Figure S12 **Integrin-β1 and Rac1 but not ROCK inhibited collective migration on a glass surface.** *(A)* The scatter plot shows the migration distance from the initial leading edge of cells deposited on a collagen coated glass substrate. The observation times correspond to the values in Fig. 3. The mean values from three independent experiments are shown. *(B)* Histogram indicating the mean ratio of the migration velocity in the presence of inhibitors. The ratio is calculated by dividing the migration velocities after and before treatment. The mean values from at least three independent experiments are shown with SD (shown as error bars), *p<0.05.
(TIF)

Movie S1 **Lumen formation by MDCK cells on a collagen gel after an overlay with another collagen gel.** Elapsed time of 1 s in the video represents 150 min of real time.
(AVI)

Movie S2 **Three-dimensional live imaging of viable MDCK cells after the collagen gel overlay.** Elapsed time of 1 s in the video represents 330 min of real time.
(AVI)

Movie S3 **Lumen formation by an “L”-shaped MDCK sheet.** The cell sheet was cut using micromanipulator and overlaid with a collagen gel. Elapsed time of 1 s in the video represents 30 min of real time.
(AVI)

Movie S4 **Lumen formation of an “O”-shaped MDCK sheet.** The cell sheet was cut using a micromanipulator and overlaid with collagen gel. Elapsed time of 1 s in the video represents 30 min of real time.
(AVI)

Movie S5 **Lumen formation of a “V”-shaped MDCK sheet.** The cell sheet was cut using a micromanipulator and overlaid with collagen gel. Elapsed time of 1 s in the video represents 30 min of real time.
(AVI)

Movie S6 **Lumen formation of an “E”-shaped MDCK sheet.** The cell sheet was cut using a micromanipulator and overlaid with the collagen gel. Elapsed time of 1 s in the video represents 30 min of real time.
(AVI)

Movie S7 **Cell movement in the presence of TGF-β1.** Cells were overlaid with a collagen gel. Elapsed time of 1 s in the video represents 150 min of real time.
(AVI)

Protocol S1 **Computational simulation.**
(DOC)

## References

[1] Lubarsky B, Krasnow MA (2003) Tube morphogenesis: Making and shaping biological tubes. Cell 112: 19–28. 12526790 10.1016/s0092-8674(02)01283-7

[2] O'Brien LE, Zegers MMP, Mostov KE (2002) Opinion - Building epithelial architecture: insights from three-dimensional culture models. Nat Rev Mol Cell Biol 3: 531–537. 12094219 10.1038/nrm859

[3] Yu W, Datta A, Leroy P, O'Brien LE, Mak G, et al (2005) beta 1-integrin orients epithelial polarity via Rac1 and laminin. Mol Biol Cell 16: 433–445. 15574881 10.1091/mbc.E04-05-0435 PMC545874

[4] Yu W, Shewan AM, Brakeman P, Eastburn DJ, Datta A, et al (2008) Involvement of RhoA, ROCK I and myosin II in inverted orientation of epithelial polarity. EMBO Rep 9: 923–929. 18660750 10.1038/embor.2008.135 PMC2529350

[5] Hall HG, Farson DA, Bissell MJ (1982) Lumen formation by epithelial-cell lines in response to collagen overlay - a morphogenetic model in culture. PNAS 79: 4672–4676. 6956885 10.1073/pnas.79.15.4672 PMC346738

[6] Schwimmer R, Ojakian GK (1995) The alpha 2 beta 1 integrin regulates collagen-mediated MDCK epithelial membrane remodeling and tubule formation. J Cell Sci 108: 2487–2498. 7673363 10.1242/jcs.108.6.2487

[7] Zuk A, Matlin KS (1996) Apical beta 1 integrin in polarized MDCK cells mediates tubulocyst formation in response to type I collagen overlay. J Cell Sci 109: 1875–1889. 8832410 10.1242/jcs.109.7.1875

[8] Eisen R, Walid S, Ratcliffe DR, Ojakian GK (2006) Regulation of epithelial tubule formation by Rho family GTPases. Am J Physiol Cell Physiol 290: C1297–C1309. 16338972 10.1152/ajpcell.00287.2005

[9] Friedl P, Hegerfeldt Y, Tusch M (2004) Collective cell migration in morphogenesis and cancer. Int J Dev Biol 48: 441–449. 15349818 10.1387/ijdb.041821pf

[10] Weijer CJ (2009) Collective cell migration in development. Journal of Cell Science 122: 3215–3223. 19726631 10.1242/jcs.036517

[11] Haga H, Irahara C, Kobayashi R, Nakagaki T, Kawabata K (2005) Collective movement of epithelial cells on a collagen gel substrate. Biophys J 88: 2250–2256. 15596493 10.1529/biophysj.104.047654 PMC1305274

[12] Omelchenko T, Vasiliev JM, Gelfand IM, Feder HH, Bonder EM (2003) Rho-dependent formation of epithelial “leader” cells during wound healing. PNAS 100: 10788–10793. 12960404 10.1073/pnas.1834401100 PMC196881

[13] Hynes RO (2002) Integrins: Bidirectional, allosteric signaling machines. Cell 110: 673–687. 12297042 10.1016/s0092-8674(02)00971-6

[14] Hall A (1994) SMALL GTP-BINDING PROTEINS AND THE REGULATION OF THE ACTIN CYTOSKELETON. Annu Rev Cell Biol 10: 31–54. 7888179 10.1146/annurev.cb.10.110194.000335

[15] Mizutani T, Haga H, Koyama Y, Takahashi M, Kawabata K (2006) Diphosphorylation of the myosin regulatory light chain enhances the tension acting on stress fibers in fibroblasts. J Cell Physio 209: 726–731. 10.1002/jcp.20773 16924661

[16] Ridley AJ (2001) Rho GTPases and cell migration. J Cell Sci 114: 2713–2722. 11683406 10.1242/jcs.114.15.2713

[17] Ishihara S, Haga H, Yasuda M, Mizutani T, Kawabata K, et al (2010) Integrin beta 1-dependent invasive migration of irradiation-tolerant human lung adenocarcinoma cells in 3D collagen matrix. BBRC 396: 651–655. 20438698 10.1016/j.bbrc.2010.04.150

[18] Wolf K, Mazo I, Leung H, Engelke K, von Andrian UH, et al (2003) Compensation mechanism in tumor cell migration: mesenchymal-amoeboid transition after blocking of pericellular proteolysis. J Cell Biol 160: 267–277. 12527751 10.1083/jcb.200209006 PMC2172637

[19] Ridley AJ (2003) Cell Migration: Integrating Signals from Front to Back. Science (New York, NY) 302: 1704–1709. 10.1126/science.1092053 14657486

[20] Hall DE, Reichardt LF, Crowley E, Holley B, Moezzi H, et al (1990) The alpha 1 beta 1-integrin and alpha 6 beta 1-integrin heterodimers mediate cell attachment to distinct sites on laminin. J Cell Biol 110: 2175–2184. 2351695 10.1083/jcb.110.6.2175 PMC2116113

[21] Hancock JF, Cadwallader K, Paterson H, Marshall CJ (1991) A CAAX OR A CAAL MOTIF AND A 2ND SIGNAL ARE SUFFICIENT FOR PLASMA-MEMBRANE TARGETING OF RAS PROTEINS. EMBO J 10: 4033–4039. 1756714 10.1002/j.1460-2075.1991.tb04979.x PMC453151

[22] Karasawa S, Araki T, Yamamoto-Hino M, Miyawaki A (2003) A Green-emitting fluorescent protein from Galaxeidae coral and its monomeric version for use in fluorescent labeling. J Biol Chem 278: 34167–34171. 12819206 10.1074/jbc.M304063200

[23] Hendzel MJ, Wei Y, Mancini MA, VanHooser A, Ranalli T, et al (1997) Mitosis-specific phosphorylation of histone H3 initiates primarily within pericentromeric heterochromatin during G2 and spreads in an ordered fashion coincident with mitotic chromosome condensation. Chromosoma 106: 348–360. 9362543 10.1007/s004120050256

[24] Xu J, Lamouille S, Derynck R (2009) TGF-beta-induced epithelial to mesenchymal transition. Cell Res 19: 156–172. 19153598 10.1038/cr.2009.5 PMC4720263

[25] Eiraku M, Takata N, Ishibashi H, Kawada M, Sakakura E, et al (2011) Self-organizing optic-cup morphogenesis in three-dimensional culture. Nature 472: 51–U73. 21475194 10.1038/nature09941

[26] Heisenberg C-P, Bellaiche Y (2013) Forces in Tissue Morphogenesis and Patterning. Cell 153: 948–962. 23706734 10.1016/j.cell.2013.05.008

[27] Reffay M, Parrini MC, Cochet-Escartin O, Ladoux B, Buguin A, et al (2014) Interplay of RhoA and mechanical forces in collective cell migration driven by leader cells. Nat Cell Biol 16: 217-+. 24561621 10.1038/ncb2917

[28] Hellman NE, SpectorO J, Robinson J, Zuo X, Saunier S, et al (2008) Matrix metalloproteinase 13 (MMP13) and tissue inhibitor of matrix metalloproteinase 1 (TIMP1), regulated by the MAPK pathway, are both necessary for Madin-Darby canine kidney tubulogenesis. J Biol Chem 283: 4272–4282. 18039671 10.1074/jbc.M708027200

[29] Kadono Y, Shibahara K, Namiki M, Watanabe Y, Seiki M, et al (1998) Membrane type 1 matrix metalloproteinase is involved in the formation of hepatocyte growth factor scatter factor induced branching tubules in Madin-Darby canine kidney epithelial cells. BBRC 251: 681–687. 9790969 10.1006/bbrc.1998.9531

[30] Kivela-Rajamaki M, Maisi P, Srinivas R, Tervahartiala T, Teronen O, et al (2003) Levels and molecular forms of MMP-7 (matrilysin-1) and MMP-8 (collagenase-2) in diseased human peri-implant sulcular fluid. J Periodontal Res 38: 583–590. 14632921 10.1034/j.1600-0765.2003.00688.x

[31] Ishihara S, Yasuda M, Harada I, Mizutani T, Kawabata K, et al (2013) Substrate stiffness regulates temporary NF-kappa B activation via actomyosin contractions. Exp Cell Res 319: 2916–2927. 24113574 10.1016/j.yexcr.2013.09.018

[32] Butcher DT, Alliston T, Weaver VM (2009) A tense situation: forcing tumour progression. Nat. Rev. Cancer 9: 108–122. 10.1038/nrc2544 PMC2649117 19165226

[33] Yamao M, Naoki H, Ishii S (2011) Multi-Cellular Logistics of Collective Cell Migration. Plos One 6 (12) e27950. 22205934 10.1371/journal.pone.0027950 PMC3244380

[34] Mizutani T, Haga H, Kato K, Matsuda K, Kawabata K (2006) Observation of stiff domain structure on collagen gels by wide-range scanning probe microscopy. JJAP 45: 2353–2356.

[35] Fenteany G, Janmey PA, Stossel TP (2000) Signaling pathways and cell mechanics involved in wound closure by epithelial cell sheets. Curr Biol 10: 831–838. 10899000 10.1016/s0960-9822(00)00579-0

[36] Herard AL, Pierrot D, Hinnrasky J, Kaplan H, Sheppard D, et al (1996) Fibronectin and its alpha(5)beta(1)-integrin receptor are involved in the wound-repair process of airway epithelium. Am J Physiol Lung Cell Mol Physiol 271: L726–L733. 10.1152/ajplung.1996.271.5.L726 8944715

[37] Hergott GJ, Nagai H, Kalnins VI (1993) Inhibition of retinal-pigment epithelial-cell migration and proliferation with monoclonal-antibodies against the beta-1 integrin subunit during wound-healing in organ-culture. Invest Ophthalmol Vis Sci 34: 2761–2768. 7688361

[38] Engler AJ, Sen S, Sweeney HL, Discher DE (2006) Matrix elasticity directs stem cell lineage specification. Cell 126: 677–689. 16923388 10.1016/j.cell.2006.06.044

[39] Nakayama M, Amano M, Katsumi A, Kaneko T, Kawabata S, et al (2005) Rho-kinase and myosin II activities are required for cell type and environment specific migration. Genes to Cells 10: 107–117. 15676022 10.1111/j.1365-2443.2005.00823.x

[40] Totsukawa G, Wu Y, Sasaki Y, Hartshorne DJ, Yamakita Y, et al (2004) Distinct roles of MLCK and ROCK in the regulation of membrane protrusions and focal adhesion dynamics during cell migration of fibroblasts. J Cell Biol 164: 427–439. 14757754 10.1083/jcb.200306172 PMC2172229

[41] Zahm JM, Kaplan H, Herard AL, Doriot F, Pierrot D, et al (1997) Cell migration and proliferation during the in vitro wound repair of the respiratory epithelium. Cell Motil Cytoskeleton 37: 33–43. 9142437 10.1002/(SICI)1097-0169(1997)37:1<33::AID-CM4>3.0.CO;2-I

[42] Jacinto A, Martinez-Arias A, Martin P (2001) Mechanisms of epithelial fusion and repair. Nat Cell Biol 3: E117–E123. 11331897 10.1038/35074643

[43] Aukhil I (2000) Biology of wound healing. Periodontol 2000 22: 44–50. 11276515 10.1034/j.1600-0757.2000.2220104.x

[44] Chen J, Zhang M (2013) The Par3/Par6/aPKC complex and epithelial cell polarity. Exp Cell Res 319: 1357–1364. 23535009 10.1016/j.yexcr.2013.03.021

[45] Solnica-Krezel L (2005) Conserved patterns of cell movements during vertebrate gastrulation. Curr Biol 15: R213–R228. 15797016 10.1016/j.cub.2005.03.016

[46] Marsden M, DeSimone DW (2001) Regulation of cell polarity, radial intercalation and epiboly in Xenopus: novel roles for integrin and fibronectin. Development 128: 3635–3647. 11566866 10.1242/dev.128.18.3635

