# Supplementary figures and images for: Epithelial Sheet Folding Induces Lumen Formation by Madin-Darby Canine Kidney Cells in a Collagen Gel
Source: PLoS One. 2014 Aug 29;9(8):e99655. doi: 10.1371/journal.pone.0099655 (PMC4149355; doi:10.1371/journal.pone.0099655)

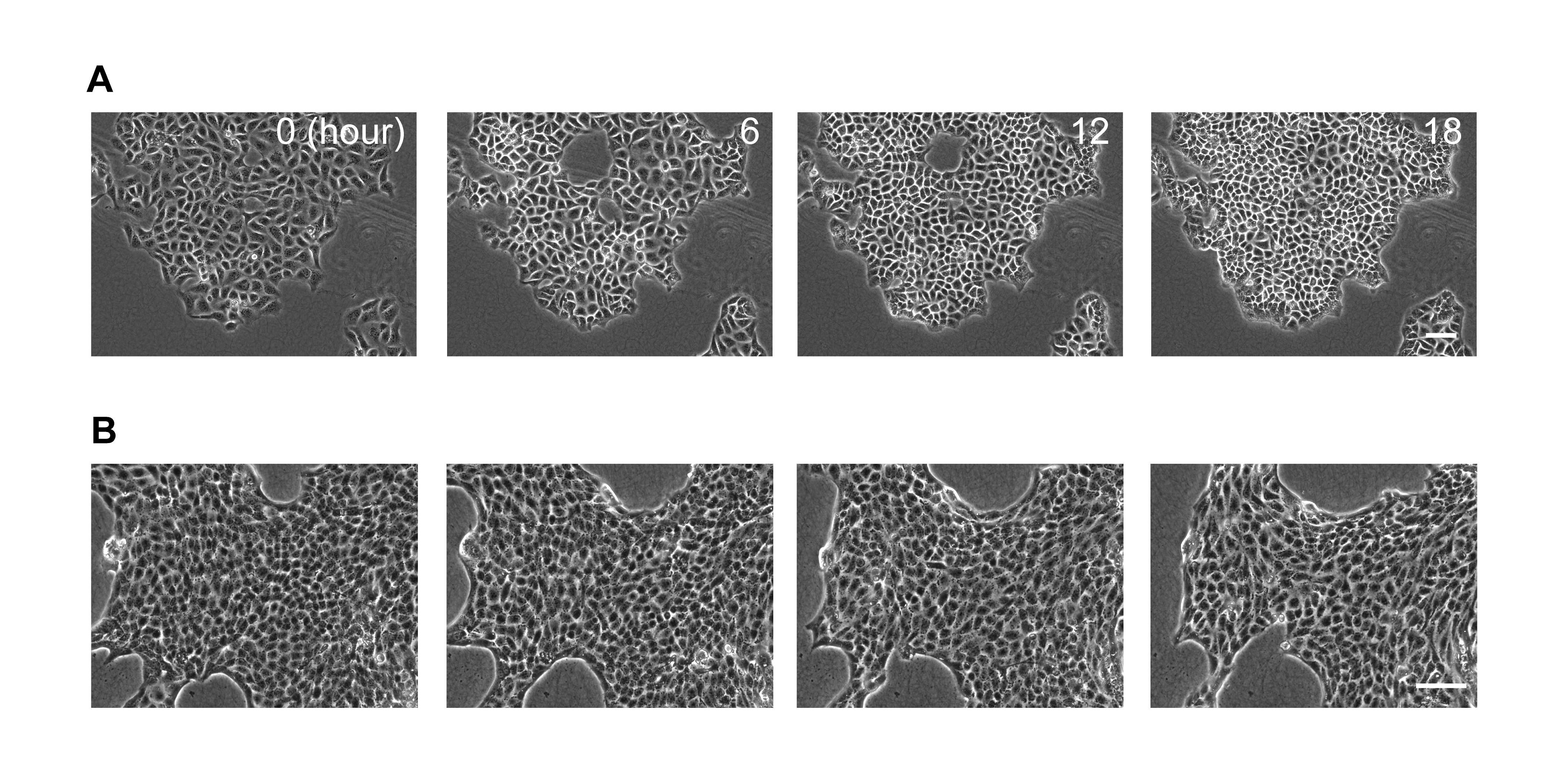

Supplement: Figure S1 — Folding movement did not occur on a glass substrate. (A) Time-lapse images of epithelial cell colonies cultured on a collagen-coated glass and overlaid with a collagen gel. Bar = 100 µm. (B) Time-lapse images of epithelial cell colonies cultured on a collagen gel and overlaid with a collagen-coated glass cover slip. Bar = 100 µm. Each observation was started immediately after the overlay. Numbers represent the relative time (h) from the start of the observation. (TIF) [file pone.0099655.s001.tif]

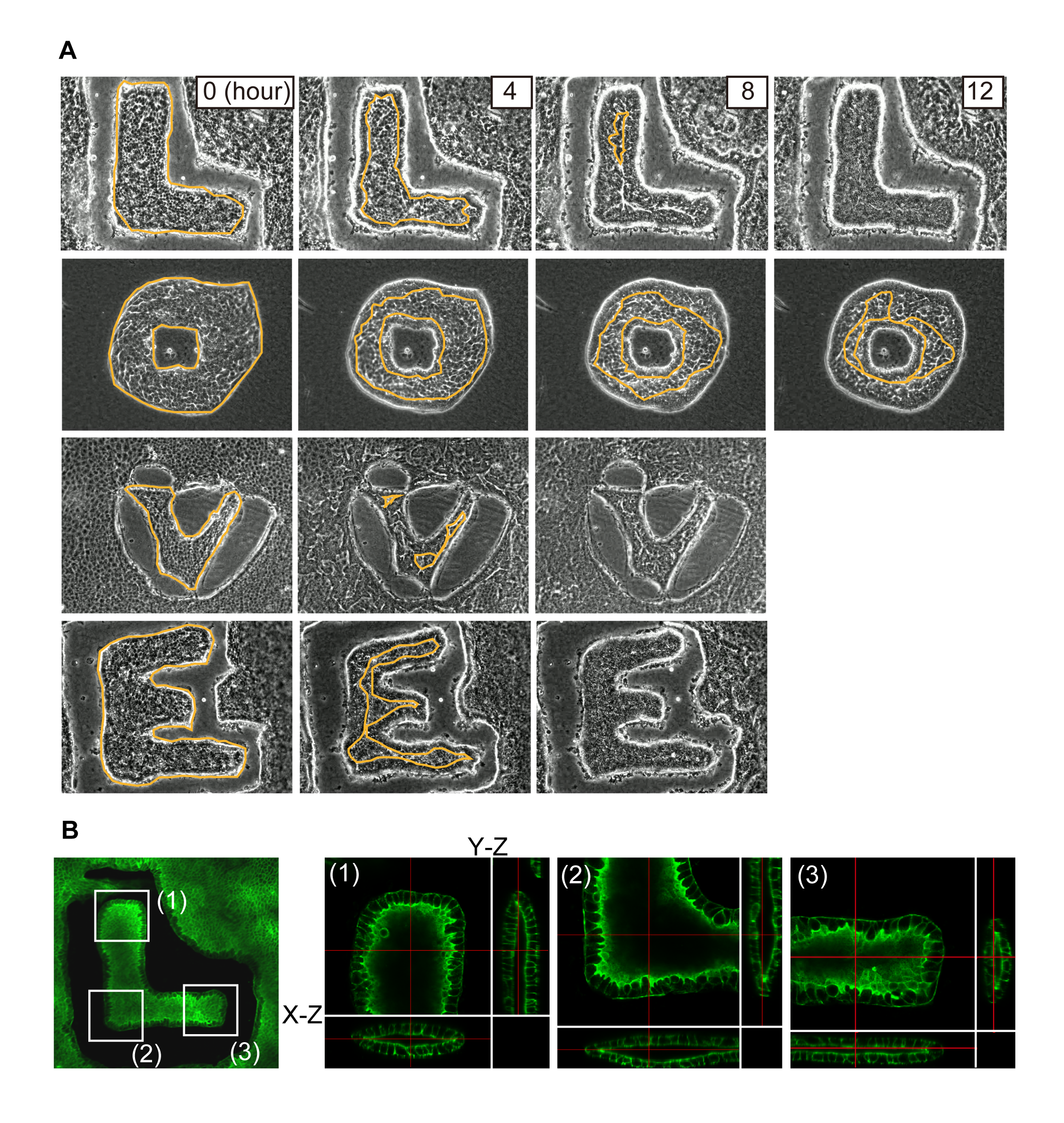

Supplement: Figure S2 — Lumen formation occurred regardless of the initial shape of the epithelial sheets. (A) Time-lapse images of epithelial sheets cut in arbitrary shapes. Each observation was started immediately after the collagen gel overlay. Orange lines represent the leading edge of the cell migration. Numbers denote the observation time (h). (B) Detection of F-actin fluorescence in the “L”-shaped structure shown in Fig. S2 A. Images (1–3) are enlargements of indicated areas in the leftmost image. (TIF) [file pone.0099655.s002.tif]

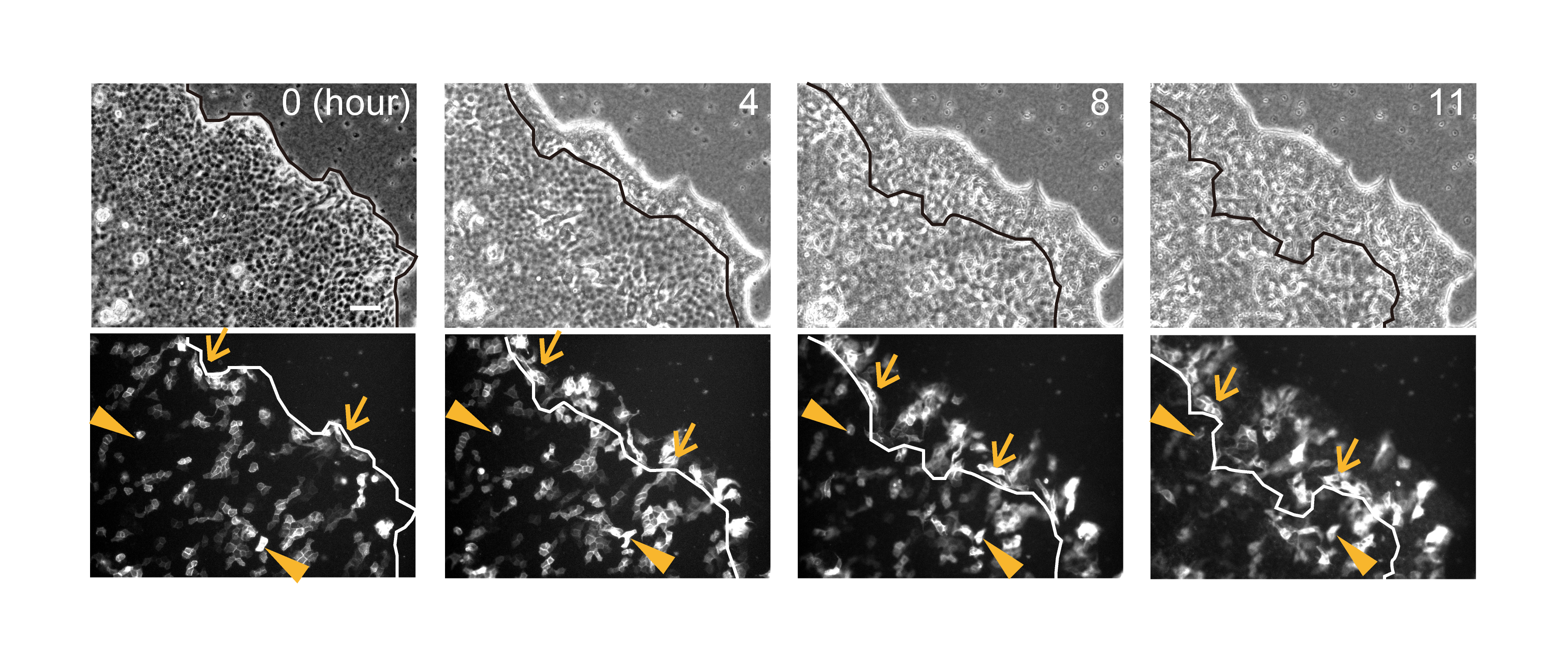

Supplement: Figure S3 — The cells near the leading edge migrated faster than those more distant. Simultaneous phase contrast (top row) and fluorescence microscopy (bottom row) of migration. Times (h) are indicated in each panel. The colony is a mosaic of fluorescent (MDCK-CAAX cells) and nonfluorescent cells. The lines represent the leading edge of the folding movement. The arrows and arrowheads chase the cells at the leading edge or within the colony, respectively. Numbers indicate the relative times (h) from the start of the observation. Bar = 100 µm. (TIF) [file pone.0099655.s003.tif]

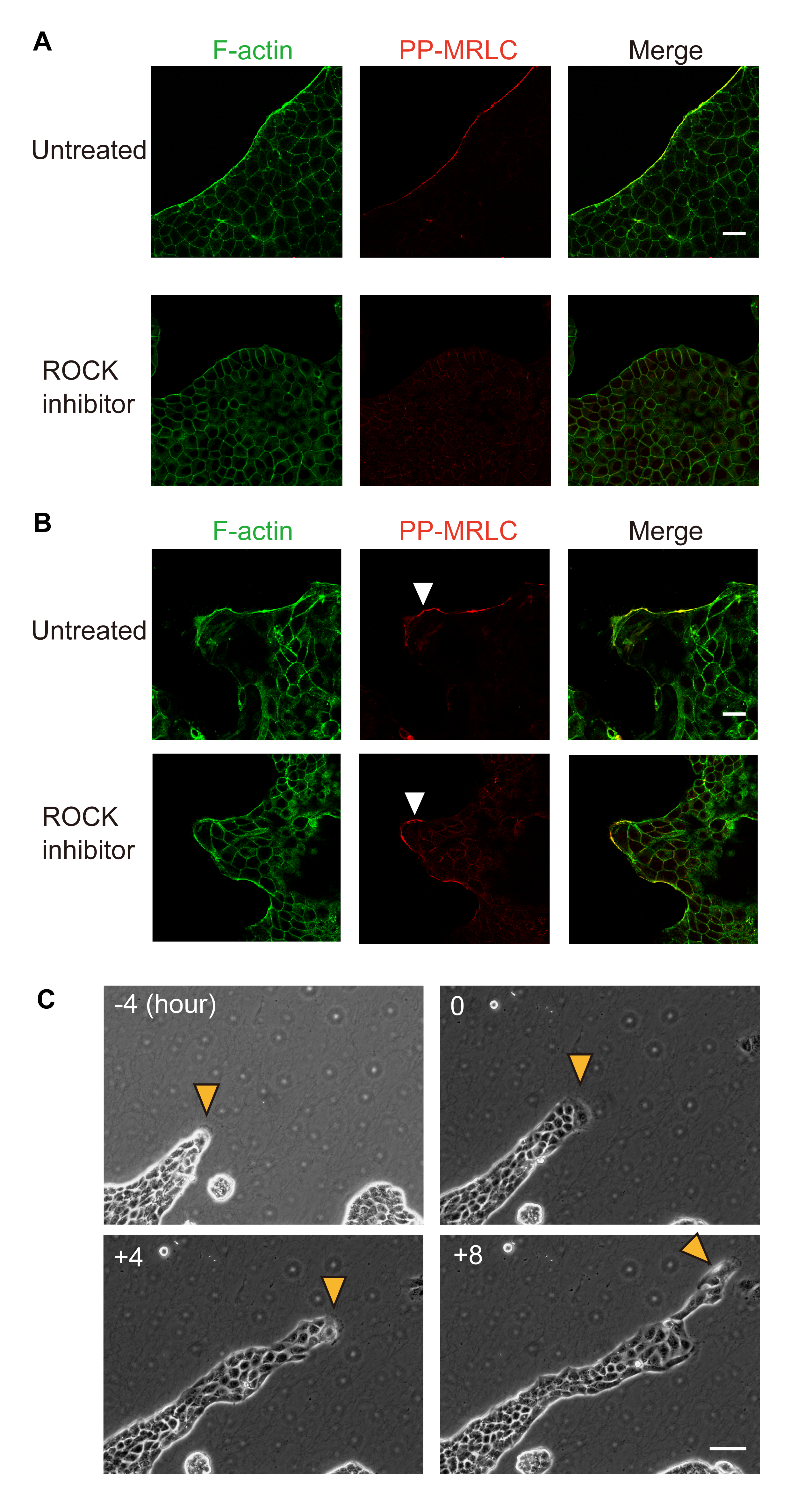

Supplement: Figure S4 — Treatment with a ROCK inhibitor induced dephosphorylation of MRLC at the edge of colonies, but in the leader cells. F-actin (green) and PP-MRLC (red) fluorescence of MDCK sheets cultured on a collagen gel. (A) Smooth edge. ROCK inhibitor (Y-27632, 10 µM) was applied for 30 min before fixation. Bar = 25 µm. (B) Leader cells. The cells were treated overnight with Y-27632 before fixation. The white arrowhead points to a leader cell. Bar = 25 µm. (C) Temporal imaging of a leader cell migrating on a collagen gel. ROCK inhibitor (Y27632, 10 µM) was added at time zero. The orange arrowheads point to the leader cells. Numbers indicate the observation time (h). Bar = 100 µm. (TIF) [file pone.0099655.s004.tif]

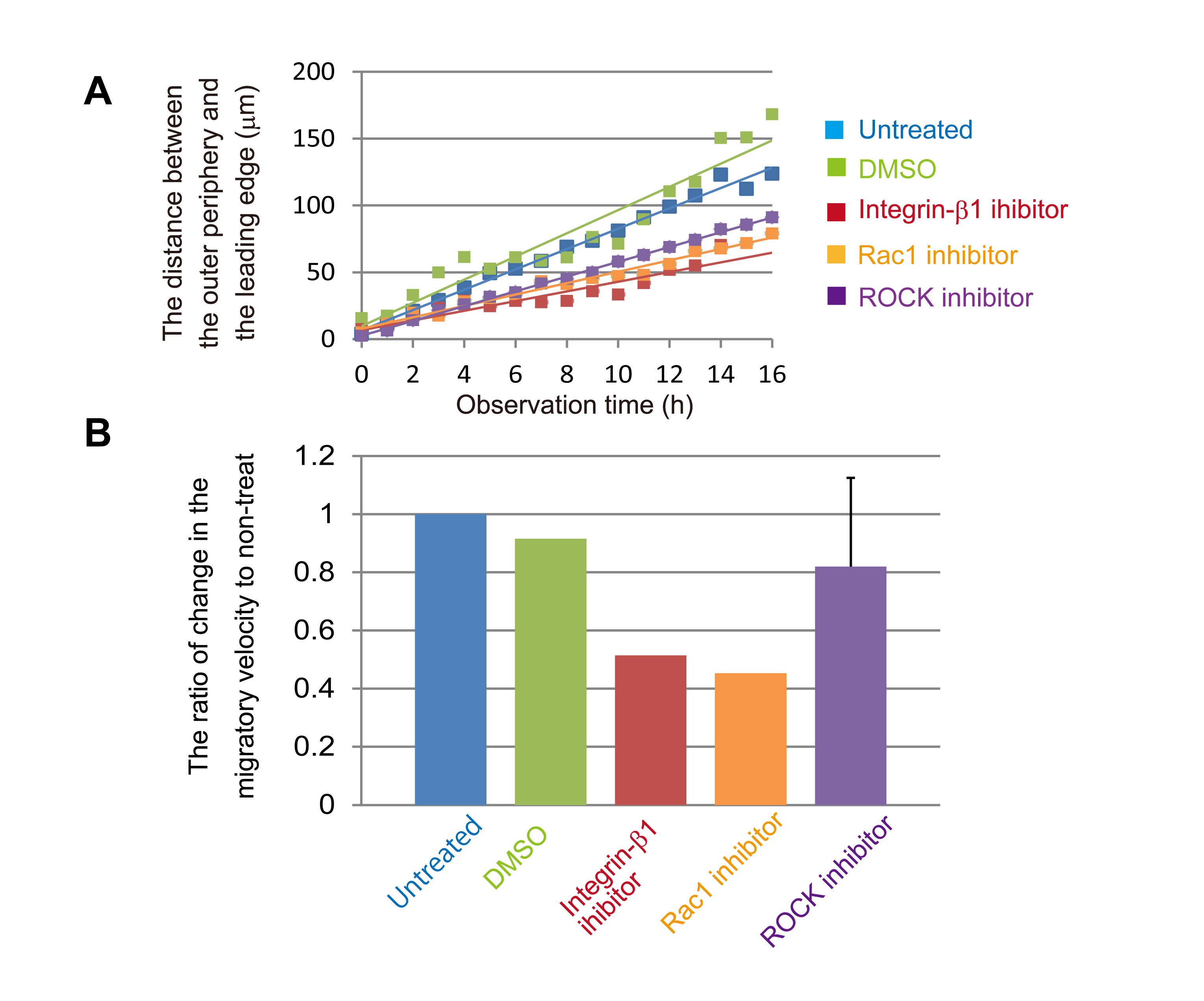

Supplement: Figure S5 — Inhibition of either integrin-β1 or Rac1 but not ROCK, delayed early folding. (A) The scatter plot shows the migration distance from the outer periphery to the leading edge for each treatment. Inhibitors were added at least 30 min before gel the overlay. The collagen solution was mixed with the indicated inhibitor and layered over the MDCK cells. Immediately after the gel formed, the observation started and continued for 16 h. The equation used to calculate the average distance is described in Materials and Methods. The mean values of at least three independent experiments are shown for untreated or cells treated with Y27632. The data acquired using the other reagents represent one experiment. (B) Histogram indicating the mean ratio of the migration velocity with or without inhibitors. The ratio is calculated by dividing the migration velocity of inhibitor-treated colonies by the velocity of untreated colonies. Shown are the mean values and SD (shown as error bars) from three independent experiments using Y27632. There was no significant difference in migration velocity between untreated and treated cells. (TIF) [file pone.0099655.s005.tif]

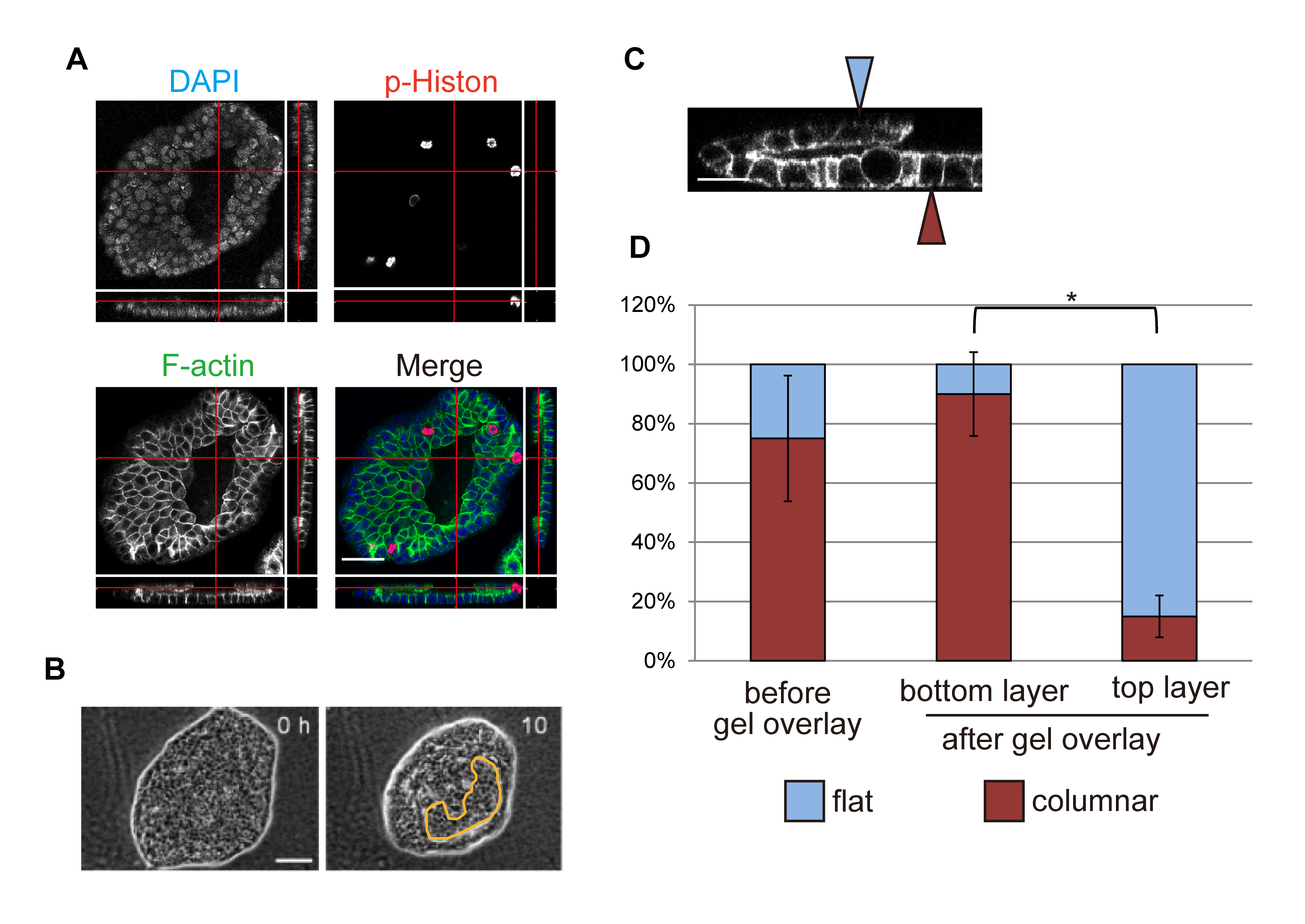

Supplement: Figure S6 — The basal area of epithelial colonies increased by cell flattening. (A) Epithelial sheets stained with DAPI (blue), and antibodies against p-histone (red) and F-actin (green) during folding. Red lines represent the planes from which the sectional views were generated. Bar = 50 µm. (B) Time-lapse imaging of roscovitine-treated (100 µM) epithelial colony after the gel overlay. Roscovitine was added immediately after the gel overlay. Numbers indicate observation times (h). The Orange line indicates the leading edge of folding. Bar = 100 µm. (C) The Z section of the image of F-actin fluorescence during folding. The blue and red arrowheads indicate flattened and columnar cells, respectively. Bar = 25 µm. (D) The ratio of flat to columnar cells in the colony before and after the gel overlay. After the gel overlay, the cell in the lower layer and those in the upper layer were counted separately. Cells were categorized as “flat” when the width was greater than height in the Z section. The mean values and SD (error bars) of 20 cells from two independent experiments; *p<0.05. (TIF) [file pone.0099655.s006.tif]

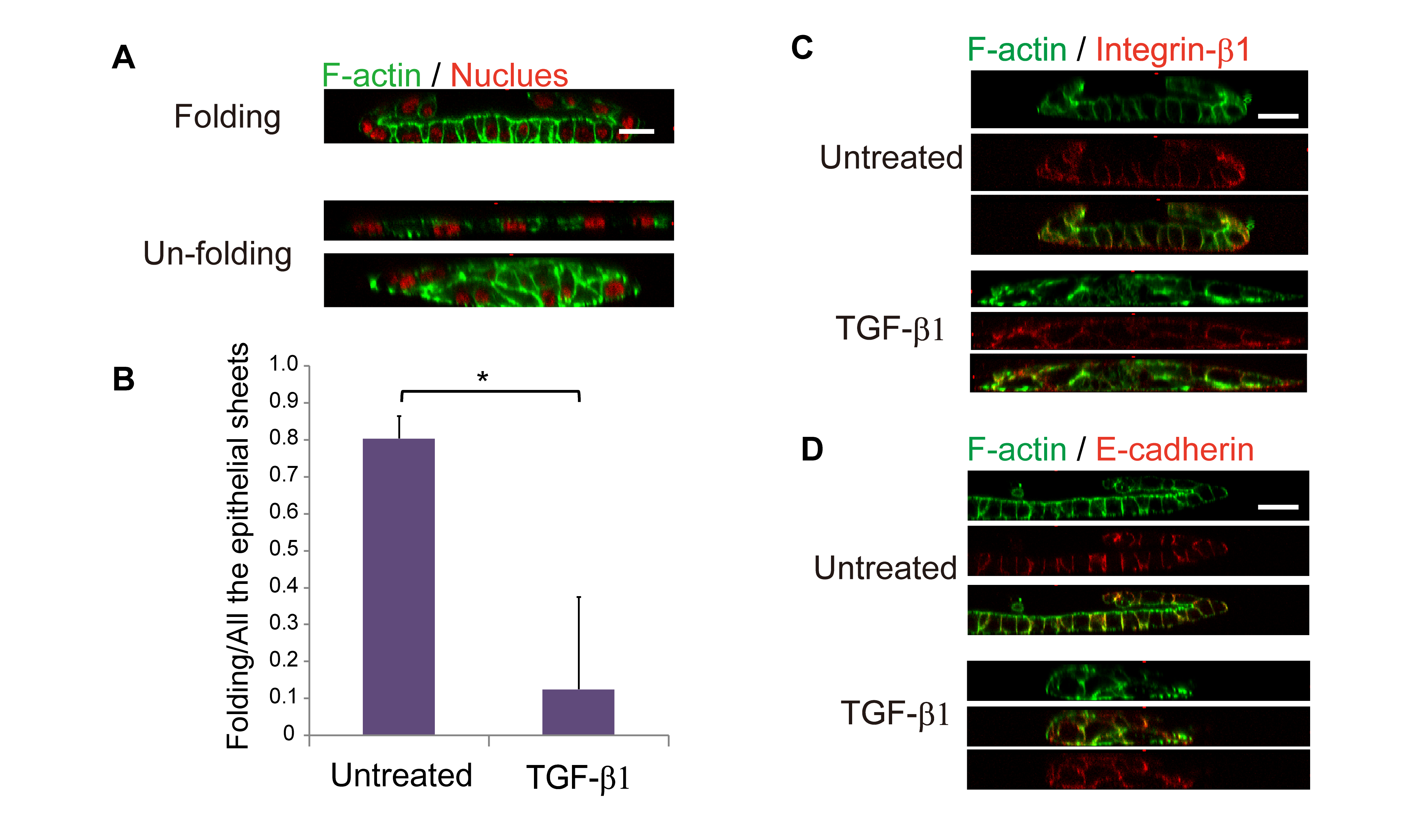

Supplement: Figure S7 — TGF-β1 treatment prevented lumen formation. (A) Categorization of folding and unfolding epithelial sheets. F-actin and nuclei were stained green and red, respectively. Cells were categorized as “folding type” when a space was observed between the upper and the lower layers of the epithelial sheet in the Z section of fluorescent images. Bar = 25 µm. (B) The ratio of folding to non-folding cells in the presence or absence of TGF-β1. The mean values are shown with SD (shown as error bars) from four independent experiments; *p<0.02. (C–D) Immunofluorescence of integrin-β1 or E-cadherin in untreated or TGF-β1-treated MDCK cells fixed 8 h after the gel overlay. The merged images with F-actin are also shown. Bar = 25 µm. (TIF) [file pone.0099655.s007.tif]

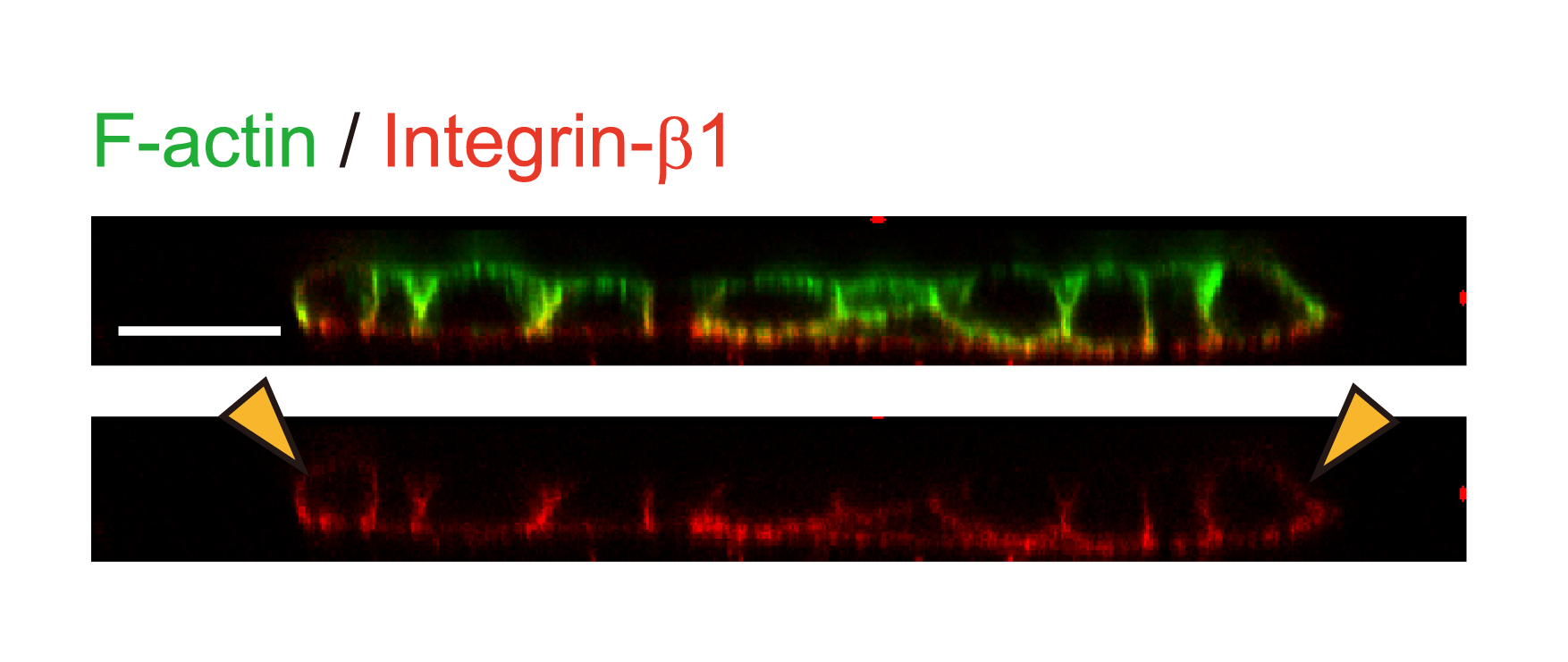

Supplement: Figure S8 — Integrin-β1 localized to the apical surface at the periphery of the MDCK colony. Integrin-β1 immunofluorescence (red) of MDCK cells on a collagen gel. The merged images with F-actin are also shown. The orange arrowheads point to the apical integrin-β1. Bar = 25 µm. (TIF) [file pone.0099655.s008.tif]

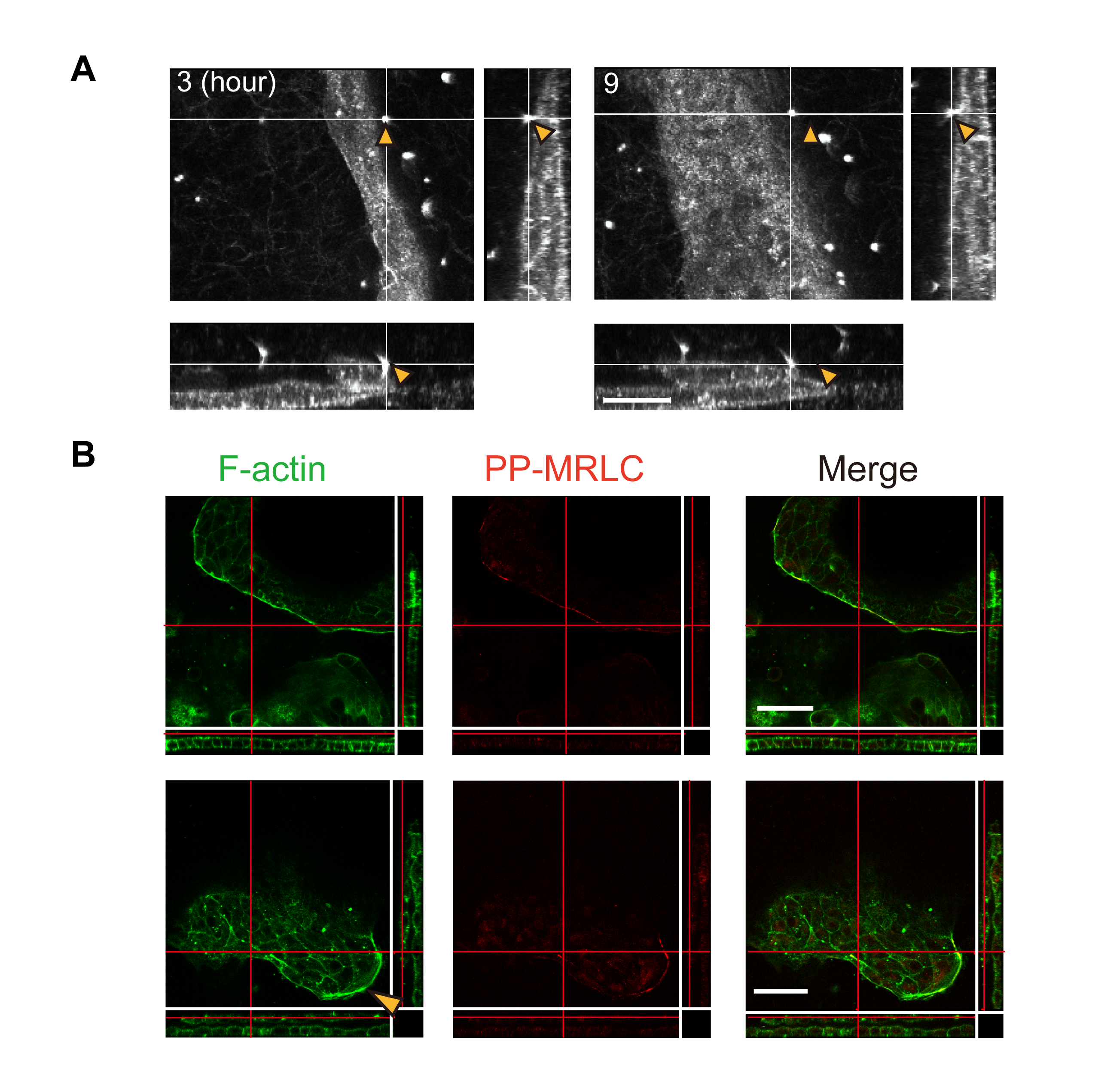

Supplement: Figure S9 — MDCK cells deformed the collagen gel during lumen formation. (A) 3D time-lapse images of MDCK cells within a latex bead-containing collagen gel. Images were acquired using the reflection interference mode of a confocal fluorescence microscope. The observation was started 30 min after the collagen gel overlay. Numbers denote the relative time from the start of the observation. The orange arrowhead points to the position of the beads at 0 h. Four beads were tracked in one experiment. Bar = 25 µm. (B) F-actin (green) and PP-MRLC (red) immunofluorescence in MDCK cells during lumen formation. Sectional views along the red lines are shown. The orange arrowhead points to a leader cell. Bar = 50 µm. (TIF) [file pone.0099655.s009.tif]

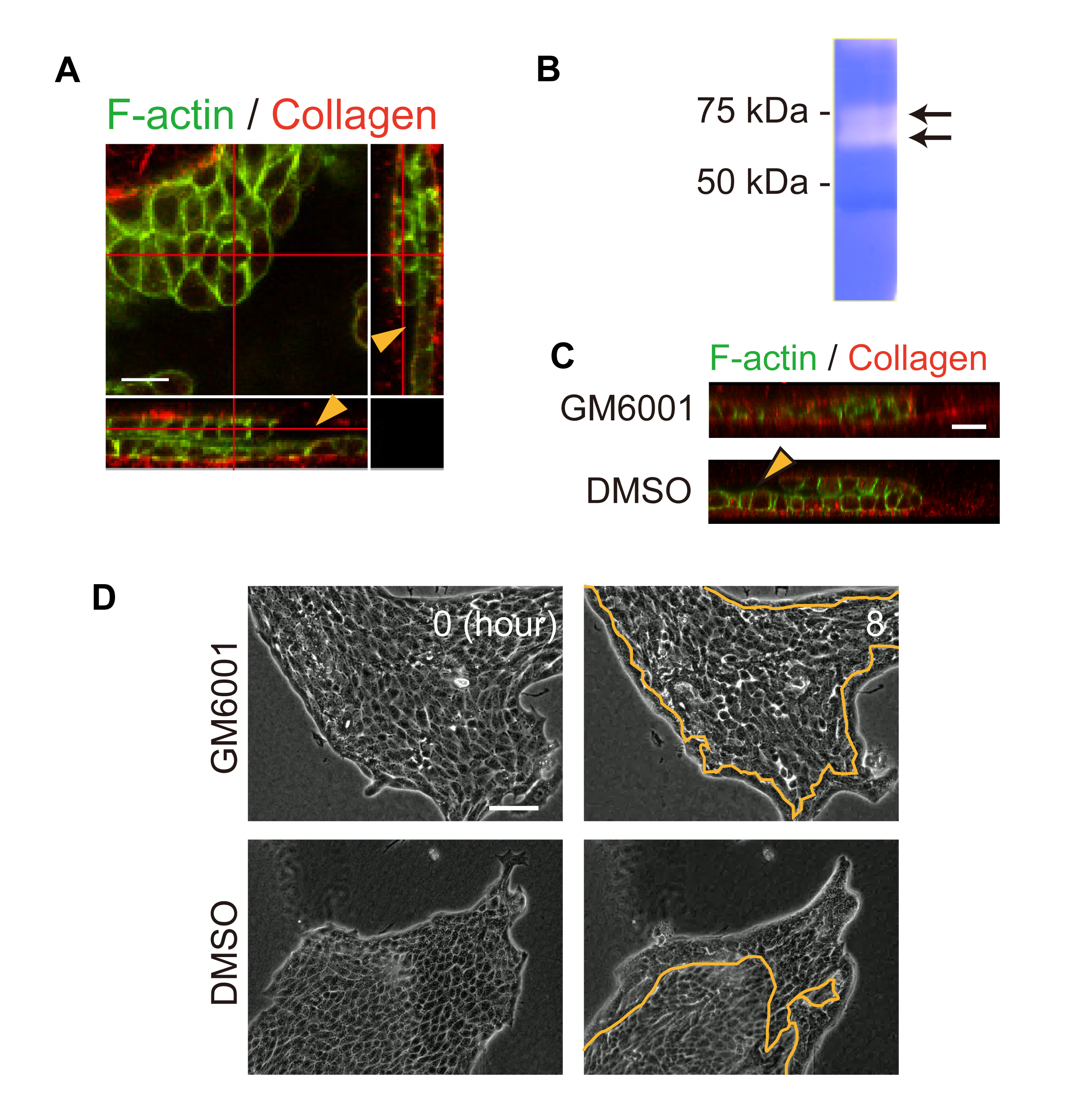

Supplement: Figure S10 — MDCK cells degraded the collagen gel. (A) Collagen (red) and F-actin (green) immunofluorescence in the MDCK colony during lumen formation. MDCK cells were fixed 6 h after the gel overlay. Red lines indicate the plane from which the sectional view was generated. Orange arrowheads point to the region between the upper collagen layer and the lower cell sheet that did not contain collagen. Bar = 20 µm. (B) Collagen zymography of the culture supernatant from MDCK cells under the gel. The black arrows point to the MMP bands that migrated at positions consistent with those of the precursor (upper) active (lower) forms of MMP-8. (C) Collagen (red) and F-actin (green) immunofluorescence in an MDCK colony treated with DMSO or GM6001 (30 nM) Z sections. The cells were fixed 15 h after the gel overlay. Orange arrowheads point to regions without the collagen gel. Bar = 25 µm. (D) Time-lapse observation of MDCK cells in the presence or absence of GM6001, which was added after the collagen gel overlay. The orange line represents the leading edge of the migrating sheet. Numbers indicate the relative time from the start of the observation. Bar = 100 µm. (TIF) [file pone.0099655.s010.tif]

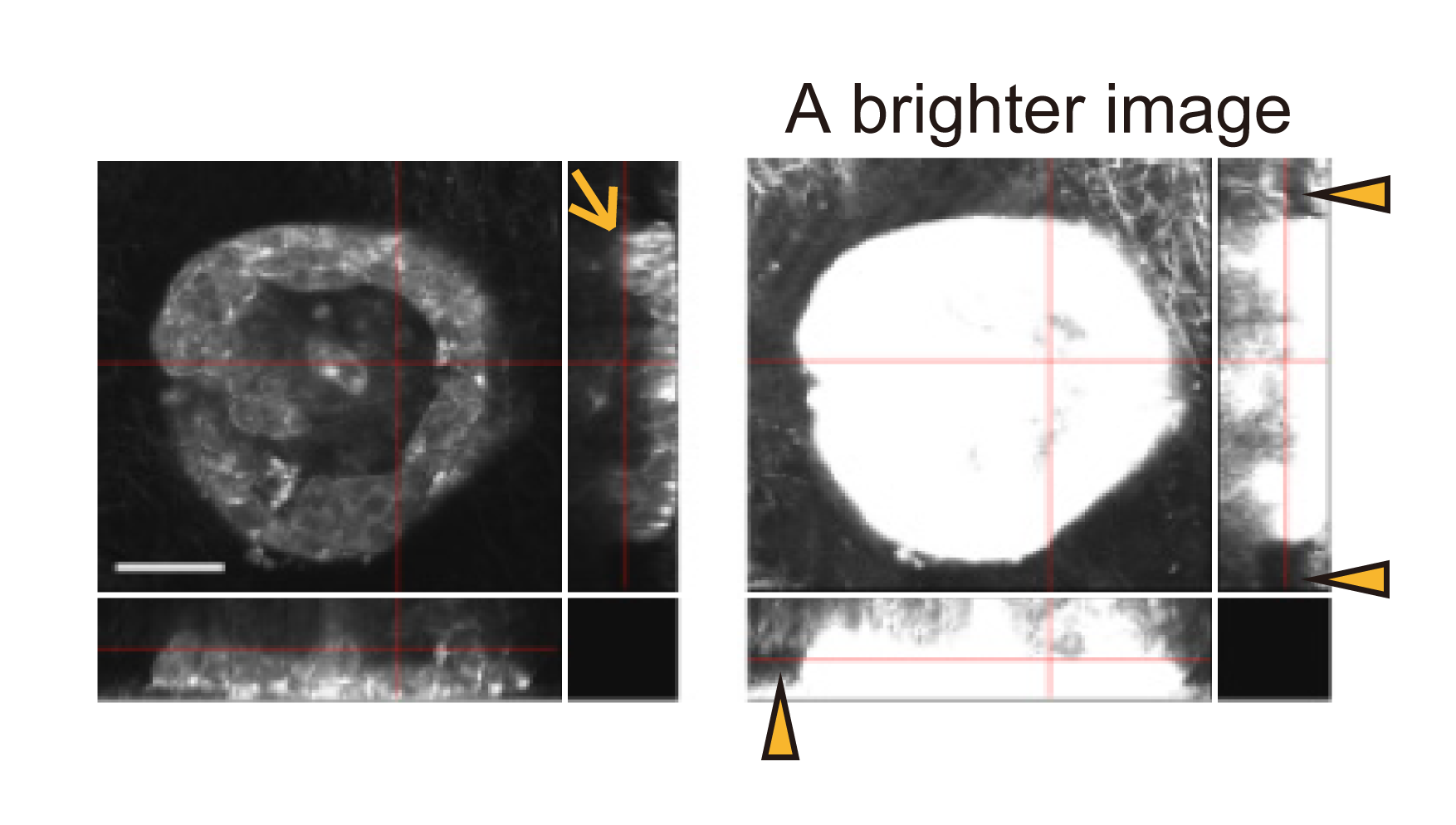

Supplement: Figure S11 — MDCK cells under the collagen gel did not migrate to collagen-gel free space. Interference reflection images of an MDCK sheet 11 hours after the gel overlay. The cells were fixed 15 h after the gel overlay. Sectional views were generated from the red lines. The orange arrow in the right column of the image on the left points to the cells that migrated to the upper layer. The image in the right panel is an overexposure. Orange arrowheads point to the collagen gel-free space between collagen layers. Bar = 50 µm. (TIF) [file pone.0099655.s011.tif]

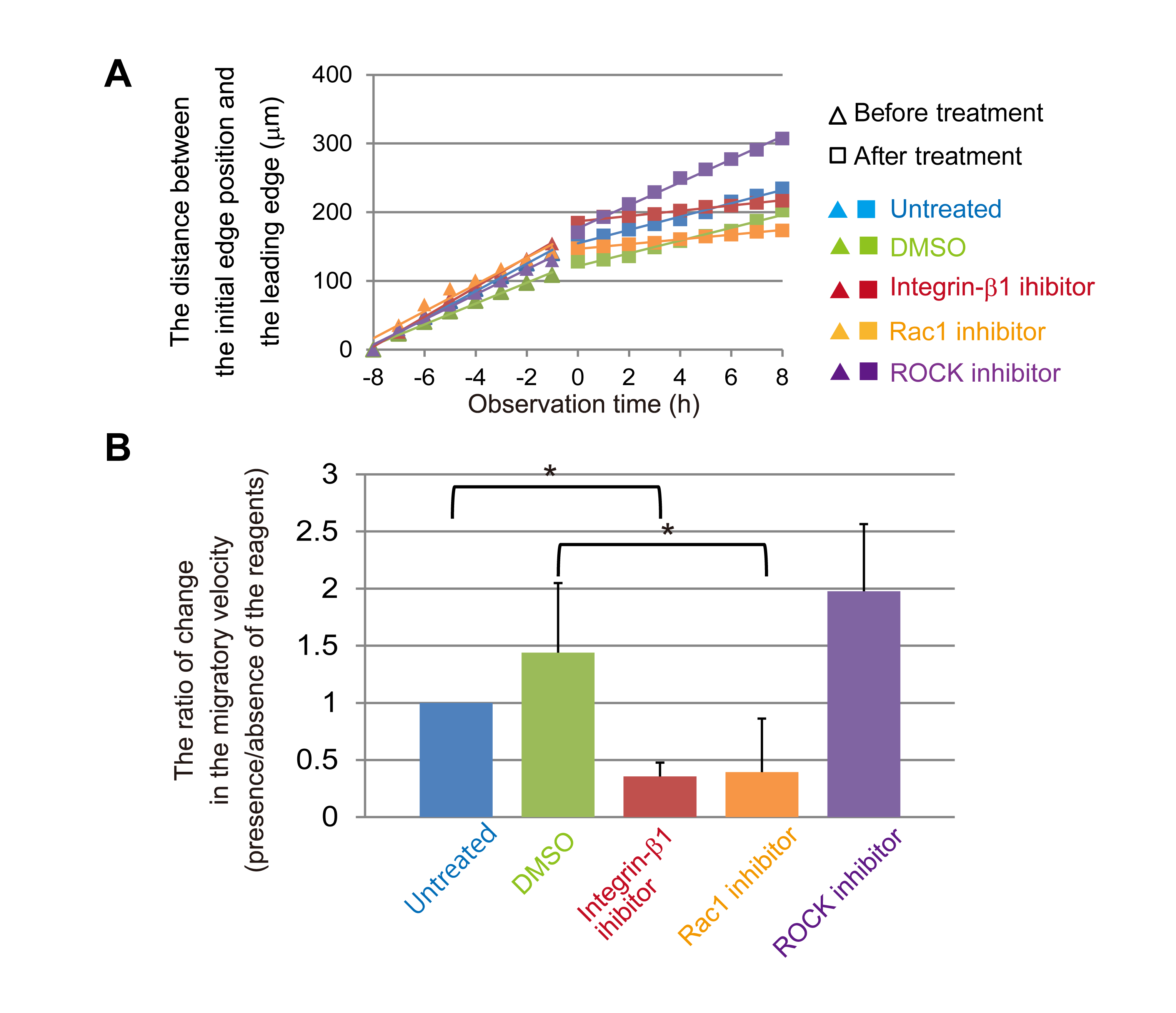

Supplement: Figure S12 — Integrin-β1 and Rac1 but not ROCK inhibited collective migration on a glass surface. (A) The scatter plot shows the migration distance from the initial leading edge of cells deposited on a collagen coated glass substrate. The observation times correspond to the values in Fig. 3. The mean values from three independent experiments are shown. (B) Histogram indicating the mean ratio of the migration velocity in the presence of inhibitors. The ratio is calculated by dividing the migration velocities after and before treatment. The mean values from at least three independent experiments are shown with SD (shown as error bars), *p<0.05. (TIF) [file pone.0099655.s012.tif]
