# Supplementary material for: Epithelial Sheet Folding Induces Lumen Formation by Madin-Darby Canine Kidney Cells in a Collagen Gel
Source: PLoS One. 2014 Aug 29;9(8):e99655. doi: 10.1371/journal.pone.0099655 (PMC4149355; doi:10.1371/journal.pone.0099655)
Supplement: Protocol S1 — Computational simulation. (DOC) [file pone.0099655.s020.doc]

**Protocol S1 Computational simulation.**

**The details of the parameters and equations**

**Fig. 6*A***: The blue circles in the chain are numbered in serial order, represented as *i* in subscripts, where *j = i +* 1*. r_ci_* represents the position of the center of the *i*^th^ blue circle (shown as a white dot in Fig. 6*A*). *R* is the radius of a circle.

**Fig. 6*B***: The two black dots have force causing random motion. The force is defined as Eq. 1 (*rand* in subscript). The parameters *r_ti_* and *r_bi_* represent the positions of the top (*t* in subscript) and the bottom (*b* in subscript) dots, respectively. The black line between the dots is the spring to connects the dots (Eqs. 2*A* and 2*B*). The parameter of the spring is represented as *con* in subscript. The dynamics of these points are described as follows:

$\mathbf{F}_{rand}=W\left(\begin{matrix} \cos\theta_{rand} \\ \sin\theta_{rand} \end{matrix}\right)$ (1)

$\mathbf{F}_{con_{tibi}}=k_{con}(L_{con}-\left|\mathbf{r}_{ti}-\mathbf{r}_{bi}\right|)\frac{\mathbf{r}_{ti}-\mathbf{r}_{bi}}{\left|\mathbf{r}_{ti}-\mathbf{r}_{bi}\right|}$ (2*A*)

$\mathbf{F}_{con_{biti}}=k_{con}(L_{con}-\left|\mathbf{r}_{bi}-\mathbf{r}_{ti}\right|)\frac{\mathbf{r}_{bi}-\mathbf{r}_{ti}}{\left|\mathbf{r}_{bi}-\mathbf{r}_{ti}\right|}$ (2*B*)

where *W* is the magnitude of force,  *θ_rand_* is an angle that is an independent random function of time, *k* is the spring constant, and *L* is the natural length of springs. *L* is equal to the diameter of a circle, except for *L_sc_* (described below).

**Fig. 6*C*:** The repulsive force is applied between the blue circles (Eq. 3, *rep* in subscript).

$\mathbf{F}_{rep_{ij}}=k_{rep}(2R-\left|\mathbf{r}_{ci}-\mathbf{r}_{cj}\right|)\frac{\mathbf{r}_{ci}-\mathbf{r}_{cj}}{\left|\mathbf{r}_{ci}-\mathbf{r}_{cj}\right|}$ (3)

This equation holds only when *2R* - |***r****_ci_* - ***r****_cj_*| > 0; otherwise, ***F****_rep ij_* = 0.

**Fig. 6*D*:** Shear tolerance, the parameter to maintain monolayer structure, is applied by the two pairs of spring. The dynamics of the parallel springs are described as Eqs. 4*A* and 4*B* (*sp* in subscript), and one of the crossed springs is described as Eqs. 5*A* and 5*B* (*sc* in subscript).

$\mathbf{F}_{sp_{titj}}=k_{sp}(L_{sp}-\left|\mathbf{r}_{ti}-\mathbf{r}_{tj}\right|)\frac{\mathbf{r}_{ti}-\mathbf{r}_{tj}}{\left|\mathbf{r}_{ti}-\mathbf{r}_{tj}\right|}$ (4*A*)

$\mathbf{F}_{sp_{bibj}}=k_{sp}(L_{sp}-\left|\mathbf{r}_{bi}-\mathbf{r}_{bj}\right|)\frac{\mathbf{r}_{bi}-\mathbf{r}_{bj}}{\left|\mathbf{r}_{bi}-\mathbf{r}_{bj}\right|}$ (4*B*)

$\mathbf{F}_{sc_{tibj}}=k_{sc}(L_{sc}-\left|\mathbf{r}_{ti}-\mathbf{r}_{bj}\right|)\frac{\mathbf{r}_{ti}-\mathbf{r}_{bj}}{\left|\mathbf{r}_{ti}-\mathbf{r}_{bj}\right|}$ (5*A*)

$\mathbf{F}_{sc_{bitj}}=k_{sc}(L_{sc}-\left|\mathbf{r}_{bi}-\mathbf{r}_{tj}\right|)\frac{\mathbf{r}_{bi}-\mathbf{r}_{tj}}{\left|\mathbf{r}_{bi}-\mathbf{r}_{tj}\right|}$ (5*B*)

where *L_sc_* is the square root of *2R*.

**Fig. 6*E*:** The elastic force from a surrounding substrate (*base* in subscript) is defined as the following equation:

(6*A*)

(6*B*)

where *B* is the initial *r_ci_*, *B_xr_* or *B_xl_* is the initial *X* coordinate of *r_ci_* of the positive or the negative edge in a chain, respectively, and *B_y_* is the initial *Z* coordinate of *r_ci_*. When *B_y_* - *r_ci y_ < 0*, Eq 6 *A* is applied. When *B_y_* - *r_ci y_ > 0*, Eq 6 *B* is applied. The elastic force ***F****_base xp_* holds only when *r_ci x_* > *B_xp_*, ***F****_base xl_* holds only when *r_ci x_* < *B_xn_*, and ***F****_base z_*holds only when *B_xn_* < *r_cix_* < *B_xp_*, respectively.

**Fig. 6*F*:** The migratory force is defined as the following equation (*mig* in subscript).

$\mathbf{F}_{mig}=\left(\begin{matrix} C \\ 0 \end{matrix}\right)$ (7)

where *C* is the magnitude of force, + ***F****_mig_* is applied to *r_ti_* of the negative edge in a chain, and -***F****_mig_* is applied to *r_ti_* of the positive edge in a chain.

Altogether, the dynamics of the circle position are described as follows:

$\mu\frac{dr_{ti}}{dt}=\sum\mathbf{F}_{rand_{ti}}+\sum\mathbf{F}_{str_{tibi}}+\sum\mathbf{F}_{rep_{cicj}}+\sum\mathbf{F}_{sp_{titj}}+\sum\mathbf{F}_{sc_{tibj}}+\sum\mathbf{F}_{base_{ti}}(\pm\mathbf{F}_{mig})$ (8*A*)

$\mu\frac{dr_{bi}}{dt}=\sum\mathbf{F}_{rand_{bi}}+\sum\mathbf{F}_{str_{biti}}+\sum\mathbf{F}_{rep_{cicj}}+\sum\mathbf{F}_{sp_{bibj}}+\sum\mathbf{F}_{sc_{bitj}}+\sum\mathbf{F}_{base_{bi}}$ (8*B*)

where *μ* is a viscous modulus.

**The parameter values used in the simulation**

Equation 1: *W* = 5

Equation 2: *k_con_* = 0.5, *L* = 2*R* = 200

Equation 3: *k_rep_* = 0.3

Equation 4: *k_sp_* = 0.04

Equation 5: *k_sc_* = 0.45

Equation 6: *k_base_* = 0.005, *k_basetop_* = 0.005 (control) or 2.0 (stiff), *k_basebottom_* = 0.005 (control) or 2.0 (stiff)

Equation 7: *C* = 5

Equation 8: *μ* = 1

The initial *X* position of *r_ci_* is 200*n* (-9 < *n* < 9), the initial *Y* position of *r_cy_* is 0.
